# Supplementary material for: Multiple risk factor control, mortality and cardiovascular events in type 2 diabetes and chronic kidney disease: a population-based cohort study
Source: BMJ Open. 2018 May 8;8(5):e019950. doi: 10.1136/bmjopen-2017-019950 (PMC5942470; doi:10.1136/bmjopen-2017-019950)

SUPPLEMENTARY MATERIAL

Figure S1. Study cohort selection

Potentially eligible participants with diabetes between 2006 and 2010 (n=279,182)

- Participants who were diagnosed with type 1 or other specific types of diabetes
- Participants who were first diagnosed with diabetes before 30 years of age
- Participants who were first prescribed insulin within 180 days of the first diagnosis

Participants with type 2 diabetes (n=233,698)

Participants with a valid serum creatinine measurement between 2006 and 2010 (n=176,311)

Participants confirmed with prevalent diabetes using data within 12 months before the index date (n=127,792)

Participants aged 40 to 79 years at index date (n=108,502)

Participants with data at least 1 year before and after index date (n=106,799)

Participants with linked data for hospital care and death registration (n=66,100)

- Participants with a diagnosis of ESRD/RRT at baseline
- Participants with index eGFR <15 mL/min/1.73 m^2

Participants with type 2 diabetes with non-ESRD at index date (n=65,861)

- Participants with missing data for smoking status, BMI, HbA1c, blood pressure and/or total cholesterol at baseline
- Participants with low or high BMI <18.5 or ≥45 kg/m^2, and HbA1c <42 mmol/mol (<6.0%), systolic blood pressure <120 mmHg, diastolic blood pressure <60 mmHg, and/or total cholesterol <3 mmol/L

Study cohort for mortality evaluation (Overall N=47,860; CKD N=11,431; non-CKD N=36,429)

- Participants with a history of CVD at baseline
- Participants who developed CHD or stroke in the first year of follow-up

Study cohort for CVD evaluation

- CHD (Overall N=35,196; CKD N=7,048; non-CKD N=28,148)
- Stroke (Overall N=35,621; CKD N=7,159; non-CKD N=28,462)

BMI, body mass index; CKD, chronic kidney disease; CHD, coronary heart disease; CVD, Cardiovascular diseases; eGFR, estimated glomerular filtration rate; ESRD, end-stage renal disease; RRT, renal replacement therapy

Table S1. Breakdown of risk factors controlled according to chronic kidney disease and a history of cardiovascular diseases

Number of risk factors controlled	HbA1c <53 mmol/mol (<7.0%)	Blood pressure <140/90 mmHg	Total cholesterol <5 mmol/L	No smoking	CKD			Non-CKD		
					Overall (N=11,431)	No CVD (N=7,216)	CVD (N=4,215)	Overall (N=36,429)	No CVD (N=28,569)	CVD (N=7,860)
0	–	–	–	–	138 (1)	87 (1)	51 (1)	806 (2)	678 (2)	128 (2)
1	Y	–	–	–	69 (0.6)	46 (0.6)	23 (0.6)	312 (1)	260 (0.9)	52 (0.7)
	–	Y	–	–	89 (0.8)	64 (0.9)	25 (0.6)	759 (2)	624 (2)	135 (2)
	–	–	Y	–	438 (4)	264 (4)	174 (4)	1,742 (5)	1,350 (5)	392 (5)
	–	–	–	Y	831 (7)	597 (8)	234 (6)	2,559 (7)	2,187 (8)	372 (5)
2	Y	Y	–	–	62 (0.5)	43 (0.6)	19 (0.5)	304 (0.8)	268 (0.9)	36 (0.5)
	Y	–	Y	–	245 (2)	152 (2)	93 (2)	794 (2)	590 (2)	204 (3)
	Y	–	–	Y	407 (4)	289 (4)	118 (3)	1,070 (3)	911 (3)	159 (2)
	–	Y	Y	–	390 (3)	215 (3)	175 (4)	1,911 (5)	1,446 (5)	465 (6)
	–	Y	–	Y	531 (5)	379 (5)	152 (4)	2,205 (6)	1,910 (7)	295 (4)
	–	–	Y	Y	2,527 (22)	1,615 (22)	912 (22)	7,004 (19)	5,477 (19)	1,527 (19)
3	Y	Y	Y	–	247 (2)	152 (2)	95 (2)	1,073 (3)	788 (3)	285 (4)
	Y	Y	–	Y	302 (3)	199 (3)	103 (2)	1,067 (3)	905 (3)	162 (2)
	Y	–	Y	Y	1,552 (14)	1,019 (14)	533 (13)	3,487 (10)	2,678 (9)	809 (10)
	–	Y	Y	Y	2,139 (19)	1,228 (17)	911 (22)	7,030 (19)	5,294 (19)	1,736 (22)
4	Y	Y	Y	Y	1,464 (13)	867 (12)	597 (14)	4,306 (12)	3,203 (11)	1,103 (14)

Y, meeting the criterion; –, not meeting the criterion

CKD, chronic kidney disease; CVD, (a history of) cardiovascular diseases

Figure S2. Relative hazards of individual risk factors controlled for mortality and cardiovascular events in (a) participants with chronic kidney disease (CKD) and (b) participants with non-CKD. Hazard ratios (HRs) for all-cause mortality and subdistribution hazard ratios (SHRs) for cardiovascular mortality, coronary heart disease, and stroke were adjusted for age, gender, CKD stage (for CKD cohort), body mass index, deprivation level, duration of diabetes, proteinuria status, a history of cardiovascular diseases (for mortality evaluation), prescribing of antidiabetic, antihypertensive, statins and antiplatelet drugs, and index year.

(a) CKD

Risk factors controlled	Adjusted HR/SHR (95% CI)	P value
**[All participants]**		
**All-cause mortality**		
HbA1c <53 mmol/mol (<7.0%)	0.86 (0.80 to 0.92)	<0.001
Blood pressure <140/90 mmHg	0.93 (0.87 to 0.99)	0.024
Total cholesterol <5 mmol/L	0.95 (0.87 to 1.04)	0.256
No smoking	0.65 (0.59 to 0.70)	<0.001
**Cardiovascular mortality**		
HbA1c <53 mmol/mol (<7.0%)	0.82 (0.75 to 0.90)	<0.001
Blood pressure <140/90 mmHg	0.89 (0.82 to 0.97)	0.006
Total cholesterol <5 mmol/L	0.93 (0.83 to 1.03)	0.159
No smoking	0.74 (0.67 to 0.83)	<0.001
**[Participants without previous CHD/stroke]**		
**Coronary heart disease**		
HbA1c <53 mmol/mol (<7.0%)	0.82 (0.73 to 0.92)	0.001
Blood pressure <140/90 mmHg	0.99 (0.89 to 1.10)	0.795
Total cholesterol <5 mmol/L	0.92 (0.80 to 1.04)	0.182
No smoking	0.98 (0.84 to 1.14)	0.779
**Stroke**		
HbA1c <53 mmol/mol (<7.0%)	0.93 (0.77 to 1.13)	0.472
Blood pressure <140/90 mmHg	0.87 (0.73 to 1.04)	0.131
Total cholesterol <5 mmol/L	0.88 (0.71 to 1.09)	0.250
No smoking	0.83 (0.65 to 1.05)	0.128

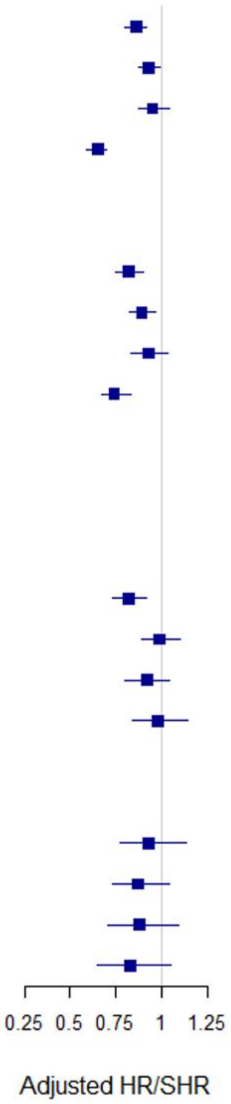

(b) Non-CKD

Risk factors controlled	Adjusted HR/SHR (95% CI)	P value
**[All participants]**		
**All-cause mortality**		
HbA1c <53 mmol/mol (<7.0%)	0.97 (0.91 to 1.03)	0.299
Blood pressure <140/90 mmHg	0.96 (0.91 to 1.02)	0.181
Total cholesterol <5 mmol/L	0.93 (0.87 to 1.00)	0.055
No smoking	0.55 (0.52 to 0.59)	<0.001
**Cardiovascular mortality**		
HbA1c <53 mmol/mol (<7.0%)	0.93 (0.85 to 1.01)	0.076
Blood pressure <140/90 mmHg	0.94 (0.87 to 1.02)	0.119
Total cholesterol <5 mmol/L	0.85 (0.77 to 0.93)	0.001
No smoking	0.58 (0.53 to 0.64)	<0.001
**[Participants without previous CHD/stroke]**		
**Coronary heart disease**		
HbA1c <53 mmol/mol (<7.0%)	0.83 (0.77 to 0.90)	<0.001
Blood pressure <140/90 mmHg	0.90 (0.84 to 0.96)	0.001
Total cholesterol <5 mmol/L	0.80 (0.74 to 0.86)	<0.001
No smoking	0.77 (0.71 to 0.83)	<0.001
**Stroke**		
HbA1c <53 mmol/mol (<7.0%)	0.92 (0.80 to 1.05)	0.207
Blood pressure <140/90 mmHg	0.77 (0.68 to 0.88)	<0.001
Total cholesterol <5 mmol/L	0.76 (0.66 to 0.88)	<0.001
No smoking	0.66 (0.57 to 0.76)	<0.001

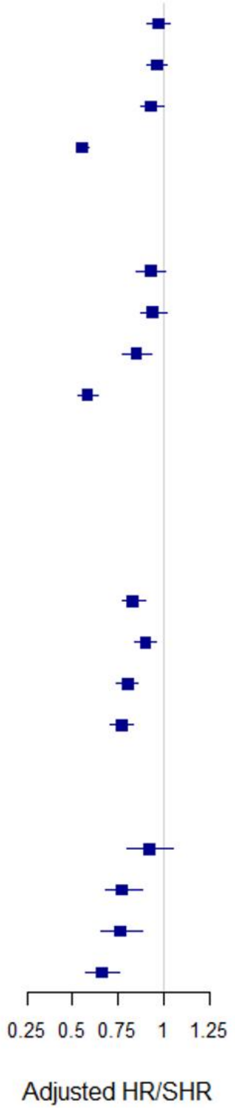

Supplement: Supplementary file 1 [file bmjopen-2017-019950supp001.pdf]
