# Supplementary material for: Multiple risk factor control, mortality and cardiovascular events in type 2 diabetes and chronic kidney disease: a population-based cohort study
Source: BMJ Open. 2018 May 8;8(5):e019950. doi: 10.1136/bmjopen-2017-019950 (PMC5942470; doi:10.1136/bmjopen-2017-019950)
Supplement: Author's manuscript [file bmjopen-2017-019950.draft_revisions.pdf]

BMJ Open

BMJ Open is committed to open peer review. As part of this commitment we make the peer review history of every article we publish publicly available.

When an article is published we post the peer reviewers' comments and the authors' responses online. We also post the versions of the paper that were used during peer review. These are the versions that the peer review comments apply to.

The versions of the paper that follow are the versions that were submitted during the peer review process. They are not the versions of record or the final published versions. They should not be cited or distributed as the published version of this manuscript.

BMJ Open is an open access journal and the full, final, typeset and author-corrected version of record of the manuscript is available on our site with no access controls, subscription charges or pay-per-view fees (http://bmjopen.bmj.com).

If you have any questions on BMJ Open's open peer review process please email info.bmjopen@bmj.com

BMJ Open

Multiple risk factor control, mortality and cardiovascular events in type 2 diabetes and chronic kidney disease: a population-based cohort study

Journal:	*BMJ Open*
Manuscript ID	bmjopen-2017-019950
Article Type:	Research
Date Submitted by the Author:	04-Oct-2017
Complete List of Authors:	Hamada, Shota; Institute for Health Economics and Policy, Research Department<br>Gulliford, Martin; King's College London, UK,
**Primary Subject Heading**:	Diabetes and endocrinology
Secondary Subject Heading:	General practice / Family practice
Keywords:	cardiovascular diseases, chronic kidney disease, mortality, type 2 diabetes

SCHOLARONE™
Manuscripts

For peer review only

Multiple risk factor control, mortality and cardiovascular events in type 2 diabetes and chronic kidney disease: a population-based cohort study

Shota Hamada[1,2], Martin C Gulliford[1,3]

1 Department of Primary Care and Public Health Sciences, King's College London, London, UK

2 Research Department, Institute for Health Economics and Policy, Association for Health Economics Research and Social Insurance and Welfare, Tokyo, Japan

3 National Institute for Health Research Biomedical Research Centre at Guy's and St Thomas' National Health Service Foundation Trust, London UK

Correspondence to: Shota Hamada

Research Department, Institute for Health Economics and Policy, Association for Health Economics Research and Social Insurance and Welfare

No.11 Toyo-kaiji Bldg, 1-5-11, Nishi-Shinbashi, Minato-ku, Tokyo, 105-0003 Japan

Tel: +81 3-3506-8529, Fax: +81 3-3506-8528, E-mail: shota.hamada@ihep.jp

For peer review only - http://bmjopen.bmj.com/site/about/guidelines.xhtml

Abstract

OBJECTIVES: This study aimed to evaluate the effectiveness of multiple risk factor control (MRFC) at reducing mortality and cardiovascular events in diabetes and chronic kidney disease (CKD) in clinical practice.

DESIGN: Population-based cohort study.

SETTING: Primary care database in the UK, linked with inpatient and mortality data.

PARTICIPANTS: Participants aged 40 to 79 years with type 2 diabetes and valid serum creatinine measurements, including 11,431 participants with CKD (eGFR 15–59 mL/min/1.73 m^2) and 36,429 participants with non-CKD (eGFR ≥60 mL/min/1.73 m^2).

EXPOSURES: MRFC consisted of four components: HbA1c <53 mmol/mol (<7.0%), blood pressure <140/90 mmHg, total cholesterol <5 mmol/L and no smoking. The main exposure variable was the number of risk factors controlled at the same time at baseline.

OUTCOME MEASURES: All-cause and cardiovascular mortality in the overall participants. Cardiovascular events, including coronary heart disease and stroke, in participants limited to those without a history of cardiovascular diseases at baseline (CKD N=7,216; non-CKD N=28,569).

RESULTS: In participants with CKD, 37% or 13% met three or four MRFC criteria, respectively. Increasing MRFC was associated with lower relative hazards for all

For peer review only - http://bmjopen.bmj.com/site/about/guidelines.xhtml

For peer review only

outcomes studied compared with those meeting no or one criterion. For participants with CKD meeting four criteria, the adjusted hazard ratio (HR) for all-cause mortality was 0.59 (95% confidence interval (CI) 0.52 to 0.67) and the adjusted subdistribution HR for cardiovascular mortality was 0.58 (0.49 to 0.69), considering a competing risk of non-cardiovascular death. Participants meeting four criteria also had lower relative hazards for coronary heart disease (adjusted subdistribution HR 0.73, 95% CI 0.59 to 0.91) and stroke (0.61, 0.43 to 0.86), considering death as a competing risk.

CONCLUSIONS: MRFC may attenuate the increased risks for mortality and cardiovascular events in people with diabetes and CKD. The implementation of MRFC is suboptimal and should be ensured in this high-risk population.

Strengths and limitations of this study

- This study included >11,000 participants with type 2 diabetes and CKD sampled from a representative general population with about 6 years of follow-up, which enabled to determine the associations of cardiovascular risk factors with mortality and cardiovascular events.
- Linked data for hospital care and death registration with a primary care database enhanced the validity of the study to evaluate mortality and cardiovascular events.

For peer review only - http://bmjopen.bmj.com/site/about/guidelines.xhtml

- We could not determine the causal relationships between MRFC and mortality and cardiovascular events from this non-randomised study.
- There is a possibility of confounding by indication; thus, healthier participants were managed more successfully and resulted in being categorised as those with greater number of risk factors controlled.

For peer review only

Introduction

Diabetes and chronic kidney disease (CKD) are growing health problems worldwide, contributing to increased mortality [1]. Diabetes and CKD also impose a substantial economic burden on society, with particularly high costs relating to cardiovascular complications and renal replacement therapy [2,3]. The prevalence of CKD in patients with diabetes is between 4.2% and 17.9% (CKD stages 3 to 5) in European countries [4]. The leading cause of death in people with type 2 diabetes or CKD is cardiovascular disease rather than renal complications [5,6]. Prevention of cardiovascular events is a key focus in the management of patients with these conditions.

Recent epidemiological studies have demonstrated additional risks of CKD on mortality and cardiovascular diseases in people with diabetes [7], but treatment approaches in this population have not been well studied. Multifactorial interventions to reduce cardiovascular risks were shown to be effective at reducing mortality and cardiovascular events in patients with type 2 diabetes and persistent microalbuminuria in the Steno-2 randomised trial [8,9]. This study provided a high level of evidence, but included a relatively small number of participants with diabetes who were managed in specialist centres. Recently, the implementation and effectiveness of this approach have been

For peer review only - http://bmjopen.bmj.com/site/about/guidelines.xhtml

evaluated in patients with diabetes in clinical practice settings [10-12]. However, no studies focused on multiple risk factor control (MRFC) in patients with both diabetes and CKD in a wide clinical practice setting. Generally, patients with kidney disease have been underrepresented in cardiovascular clinical trials [13]. This population may have an altered risk-benefit profile, and extrapolation of data based on patients with normal kidney function into patients with CKD may be unreliable [13]. We aimed to conduct a pragmatic evaluation of the effectiveness of MRFC on mortality and cardiovascular events in participants with type 2 diabetes and CKD in a population-based cohort study.

Methods

Data sources

This study employed a linked dataset derived from the UK Clinical Practice Research Datalink (CPRD), the UK National Health Service Hospital Episodes Statistics (HES) inpatient data, and the UK Office for National Statistics (ONS) mortality data. The CPRD contains anonymised electronic health records from general practices across the UK [14]. The CPRD collects data for diagnoses and clinical assessment, prescriptions and laboratory test results, such as HbA1c and serum creatinine. The HES inpatient data

For peer review only - http://bmjopen.bmj.com/site/about/guidelines.xhtml

were comprised of inpatient records from all National Health Service hospitals in England. Information on the date of death and the causes of death were available in the ONS mortality data file. Diagnoses and clinical evaluation in the CPRD were coded with the Read codes, a hierarchical coding system used in primary care in the UK, whereas those in the HES and ONS were coded with the International Classification of Diseases, tenth revision (ICD-10). Linked data are available for general practices in England only and participants were limited to those with linked data for the HES and ONS available. The study was approved by the CPRD Independent Scientific Advisory Committee (ISAC Protocol 15_201R).

Study population

The scheme of the study cohort selection is presented in figure S1. We initially sampled participants who were diagnosed with type 2 diabetes from the CPRD [15]. Using the CPRD records, the date of the first valid serum creatinine value between 2006 and 2010 recorded more than one year after the first diagnosis of diabetes were defined as the index date. A similar approach was taken by Adamsson Eryd et al [16] to ensure that participants managed for diabetes had sufficient time available for recording of baseline values. To avoid misclassification of CKD status and stage, the index serum creatinine

For peer review only - http://bmjopen.bmj.com/site/about/guidelines.xhtml

values were validated by confirmation of subsequent values within 30% of the index values. We restricted the sample to participants aged 40 to 79 years at the index date with at least one year of follow-up data available (ie, participants who died in the first year of follow-up were excluded). Estimated glomerular filtration rate (eGFR) was calculated from a serum creatinine value, age, gender, and ethnicity, using the Chronic Kidney Disease Epidemiology Collaboration (CKD-EPI) equation [17]. Missing ethnicity was assumed as 'non-black' in the present study. Participants diagnosed with end-stage renal disease, those who had received renal replacement therapy, or those with index eGFR <15 mL/min/1.73 m^2 were excluded. We also excluded participants with missing data for smoking status, body mass index (BMI), HbA1c, blood pressure, total cholesterol or with extreme BMI (<18.5 or ≥45 kg/m^2) at baseline. Since it has been reported that low values of cardiovascular risk factors were not always associated with better outcomes in observational studies [15,18,19], possibly due to reverse causation [20,21], participants with low HbA1c (<42 mmol/mol or <6.0%), blood pressure (systolic <120 or diastolic <60 mmHg) and total cholesterol (<3 mmol/L) were further excluded. Participants were categorised according to index eGFR into those with CKD (<60 mL/min/1.73 m^2) and non-CKD (≥60 mL/min/1.73 m^2).

For peer review only - http://bmjopen.bmj.com/site/about/guidelines.xhtml

Multiple risk factor control

MRFC was defined in this study as consisting of four components: (1) HbA1c <53 mmol/mol (<7.0%), (2) blood pressure <140/90 mmHg (systolic <140 and diastolic <90 mmHg), (3) total cholesterol <5 mmol/L and (4) no smoking (non- or ex-smokers). The means of HbA1c, blood pressure, and total cholesterol records within one year before the index date were evaluated. The number of the risk factors controlled from four criteria was treated as the exposure and included as a categorical variable in the analyses, with those meeting no or one criterion as a reference category.

Outcomes

Main outcomes of interest in this study included all-cause and cardiovascular mortality, fatal and non-fatal coronary heart disease (CHD) and stroke. The date of death and causes of death were determined using the ONS mortality data. Patients who died from cardiovascular causes were identified if people had any of the ICD-10 codes I00 to I99 as a cause of death. All of the CPRD, HES and ONS were used to ascertain fatal and non-fatal CHD and stroke. Read codes for CHD and stroke reported previously [22,23] were updated for the present study. The ICD-10 codes for CHD and stroke were I20 to I25 and I60, I61, I63 and I64, respectively.

For peer review only - http://bmjopen.bmj.com/site/about/guidelines.xhtml

Analysis

Baseline characteristics of the study cohort were described according to CKD status. Time-to-event analyses were conducted to evaluate the associations of MRFC with mortality and cardiovascular events. To address the issue of reverse causation and to avoid misclassification of the outcomes from those which had existed at baseline, person-years for participants who experienced outcomes of interest in the first year of follow-up were excluded from analyses. Cox proportional hazards models were used to evaluate the association of MRFC with all-cause mortality. Proportional hazards assumption was assessed by visual inspection of log-log plots, and no apparent violation was found. Competing risks regression with subdistribution hazard models were conducted for cardiovascular mortality and cardiovascular events, considering competing risks for non-cardiovascular and all-cause death, respectively [24]. Associations of MRFC with cardiovascular events were evaluated in participants without a known history of cardiovascular diseases at baseline. Participants were followed from the index date until the earliest of the events of interest, the last date of CPRD records, or 31 March 2015 for all-cause mortality evaluation. In the competing risks regression analyses for cardiovascular mortality and cardiovascular events,

For peer review only - http://bmjopen.bmj.com/site/about/guidelines.xhtml

participants who experienced the corresponding competing events prior to the event of interest were also censored.

Main analyses were conducted by CKD status, adjusting for a range of baseline covariates, including age (continuous), gender (male or female), CKD stage (3a, 3b and 4; for CKD cohort), BMI (18.5–24.9, 25.0–29.9, 30.0–34.9, 35.0–39.9 and 40.0–44.9 kg/m^2), deprivation level (quintile; 1, least deprived, to 5, most deprived), duration of diabetes (1.0–4.9 5.0–9.9 and 10+ years), a history of cardiovascular diseases, including CHD and stroke, and prescribing during six months prior to the index date of antidiabetic drugs (none, insulin with and without other antidiabetic drugs, and non-insulin drugs only), antihypertensive drugs (none, drugs acting on renin-angiotensin system with and without other antihypertensive drugs, and other classes of antihypertensive drugs only, including β-blockers, calcium channel blockers, and thiazide diuretics), statins and antiplatelet drugs, and index year (2006 to 2010). In addition, the association of CKD with the outcomes were evaluated according to the number of risk factors controlled, adjusting for the potential confounding factors described above.

For peer review only - http://bmjopen.bmj.com/site/about/guidelines.xhtml

In this paper, the results for participants with CKD were focused on, with the results for those with non-CKD shown for comparative purposes. All analyses were performed using Stata version 14 (Stata Corp., College Station TX). The 'forestplot' package in R was used to present the results [25].

Results

Characteristics of the study population

Baseline characteristics of the study cohort are shown according to CKD status in table 1. Mean index eGFR was 49 mL/min/1.73 m^2 for participants with CKD and 81 mL/min/1.73 m^2 for those with non-CKD. Participants with CKD were older (71 *vs* 62 years), included more women (52% *vs* 40%), had a longer duration of diabetes, and were more likely to have a history of cardiovascular diseases (37% *vs* 22%). HbA1c and total cholesterol were slightly lower in participants with CKD. Although diastolic blood pressure was lower in participants with CKD, systolic blood pressure was higher despite more people under antihypertensive medications. Participants with CKD were prescribed insulin, drugs on renin-angiotensin systems, statins, and antipatelet drugs more frequently.

For peer review only - http://bmjopen.bmj.com/site/about/guidelines.xhtml

For peer review only

Implementation of MRFC

The number of risk factors controlled from four components of MRFC are shown in table 2. More detailed results of which of the components were controlled are available in table S1. Higher rates of control for HbA1c, total cholesterol and smoking status were observed in participants with CKD compared with those with non-CKD. However, blood pressure was less likely managed in participants with CKD (46% *vs* 51%). There were some differences in management status according to a history of cardiovascular diseases: in participants with CKD, higher rates of control of blood pressure (49% *vs* 44%) and total cholesterol (83% *vs* 76%) in participants with a history of cardiovascular diseases compared with those without. Participants meeting three or four criteria accounted for 37% or 13% in participants with CKD.

Effectiveness of MRFC

Absolute risks for mortality and cardiovascular diseases and adjusted relative hazards of the number of risk factors controlled for the outcomes are shown in figure 1. Increasing MRFC was associated with lower relative hazards for all outcomes studied relative to participants meeting no or one criterion. For participants with CKD meeting four MRFC criteria, the adjusted hazard ratio (HR) for all-cause mortality was 0.59 (95% CI 0.52 to

For peer review only - http://bmjopen.bmj.com/site/about/guidelines.xhtml

For peer review only

0.67) and adjusted subdistribution HR for cardiovascular mortality was 0.58 (0.49 to 0.69). Participants meeting four criteria also had lower relative risks for CHD (adjusted subdistribution HR 0.73, 95% CI 0.59 to 0.91) and stroke (0.61, 0.43 to 0.86) in participants with CKD. In participants with non-CKD, increasing MRFC was also associated with lower risks for all-cause and cardiovascular mortality, CHD and stroke.

Comparisons between CKD and non-CKD

Unadjusted absolute risks for mortality and cardiovascular diseases were higher in participants with CKD by 1.4- to 2.9-fold compared with those with non-CKD at the same MRFC category (figure 1). More participants with CKD died from cardiovascular causes compared with those without (63% *vs* 54%, P<0.001). Relative hazards of CKD for the outcomes are shown in figure 2. After adjustment with possible confounding factors, comorbid CKD remained to be associated with greater risks for all-cause mortality (adjusted HR, 1.18 to 1.32), cardiovascular mortality (adjusted subdistribution HR, 1.28 to 1.44) and CHD (1.08 to 1.25). However, the associations of comorbid CKD with stroke was observed in participants meeting four criteria only (1.64).

Discussion

For peer review only
For peer review only - http://bmjopen.bmj.com/site/about/guidelines.xhtml

In this population-based cohort study of 11,431 participants with type 2 diabetes and CKD stages 3 to 4, MRFC was associated with lower relative risks for mortality and cardiovascular diseases. We also confirmed that CKD was associated with increased risks for mortality and cardiovascular events. Higher absolute risks for mortality and cardiovascular events and great relative risk reduction associated with MRFC suggest that the MRFC strategy may be one of the main approaches to potentially reducing the burden of diabetes and CKD. Nevertheless, we found that the implementation of MRFC in patients with diabetes was suboptimal in the clinical practice setting, as reported in previous studies [10-12].

This study evaluated the effectiveness of MRFC in patients with type 2 diabetes according to presence or absence of CKD in clinical practice. So far, the associations of MRFC with lower risks for mortality and cardiovascular events have been shown in people with diabetes, not focusing on CKD status. Participants with controlled three risk factors of HbA1c, blood pressure and LDL cholesterol had 62% and 60% risk reduction for cardiovascular events and CHD, respectively, in patients with diabetes without known cardiovascular diseases [11]. The associations of uncontrolled HbA1c, blood pressure, LDL cholesterol and smoking with mortality and cardiovascular events were

For peer review only - http://bmjopen.bmj.com/site/about/guidelines.xhtml

individually evaluated in a large population-based study with >850,000 participants with diabetes [12]. The study cohort included 35.5% of CKD in those with cardiovascular diseases and 21.8% in those without, and CKD was included in the analyses for adjustment. This study suggested that uncontrolled risk factors attributed to about 1 in 3 major cardiovascular events and fewer 1 in 10 deaths.

The strength of this study was the inclusion of a large size of >11,000 participants with diabetes and CKD with an observation of >62,000 person-years. In addition to the large sample size and long-term follow-up, representativeness from general population and data quality are also advantages of the CPRD [14], which should remain even if linked data for HES and ONS are only available for England practices. Instead, linked data for hospital care and death registration substantially enhanced the validity of the study to evaluate mortality and cardiovascular events.

There are also some limitations in this study. First, despite our focus on the number of MRFC, the impact of each of the risks factor on mortality and cardiovascular events should be different. Second, we could not determine the causal relationships between MRFC and mortality and cardiovascular events from this non-randomised study. Third,

For peer review only - http://bmjopen.bmj.com/site/about/guidelines.xhtml

there is a possibility of confounding by indication; thus, healthier participants were managed more successfully and resulted in being categorised as those with greater number of risk factors controlled. For example, stringent management of HbA1c might not be targeted for vulnerable participants due to concerns for greater risk of hypoglycaemia. Fourth, we cannot exclude the possibility of residual confounding despite adjustment with a range of covariates in the analyses, including physical activity and alcohol intake [26,27]. Albuminuria, not always available in our study, has been known as a risk factor for mortality and cardiovascular diseases [28,29]. A recent study suggested, however, that proteinuria status might not have substantial impact on cardiovascular outcomes in patients with diabetes and CKD [30]. Fifth, measurement and assay methods for HbA1c, blood pressure, cholesterol and serum creatinine might not have been standardised among general practices or laboratories. As well as missing data on ethnicity and fluctuations in serum creatinine values, these methodological limitations might influence the determination of CKD status or staging. Finally, although we used one of the largest primary care electronic health records database, it seemed to be insufficient to separately evaluate MRFC for participants with different stages of CKD. Further research is needed to focus on patients with more advanced CKD who may have altered risk-benefit profile compared with patients with less

For peer review only - http://bmjopen.bmj.com/site/about/guidelines.xhtml

impaired renal function.

In summary, based on the population-based cohort study of routine clinical practices, MRFC may attenuate the increased risks for mortality and cardiovascular events in people with diabetes and CKD. However, the implementation of MRFC is suboptimal, and further research is needed to clarify underlying reasons to ensure more improved achievement of MRFC in this population.

Acknowledgments

SH was supported by the Uehara Memorial Foundation Postdoctoral Fellowship, Tokyo, Japan. MCG was supported by the National Institute for Health Research (NIHR) Biomedical Research Centre at Guy's and St Thomas' NHS Foundation Trust and King's College London. This study is based on data from the CPRD obtained under license from the UK Medicines and Healthcare products Regulatory Agency. However, the interpretation and conclusions contained in this report are those of the authors alone and not necessarily those of the National Health Service, the NIHR or the Department of Health.

For peer review only - http://bmjopen.bmj.com/site/about/guidelines.xhtml

Contributors: Both authors contributed to conception and study design of the study, data acquisition, statistical analysis, and interpretation. SH drafted the manuscript and MCG revised it critically for important intellectual content.

Funding: This research received no specific grant from any funding agency.

Competing interests: None declared.

Provenance and peer review: Not commissioned; externally peer reviewed.

Data sharing statement: No additional data are available.

References

1. GBD 2013 Mortality and Causes of Death Collaborators. Global, regional, and national age-sex specific all-cause and cause-specific mortality for 240 causes of death, 1990-2013: a systematic analysis for the Global Burden of Disease Study 2013. *Lancet.* 2015;385(9963):117-171.
2. Seuring T, Archangelidi O, Suhrcke M. The Economic Costs of Type 2 Diabetes: A Global Systematic Review. *Pharmacoeconomics.* 2015;33(8):811-831.
3. Kerr M, Bray B, Medcalf J, O'Donoghue DJ, Matthews B. Estimating the financial cost of chronic kidney disease to the NHS in England. *Nephrol Dial Transplant.* 2012;27 Suppl 3:iii73-80.

For peer review only - http://bmjopen.bmj.com/site/about/guidelines.xhtml

4. Brück K, Stel VS, Gambaro G, Hallan S, Völzke H, Ärnlöv J, et al. CKD Prevalence Varies across the European General Population. *J Am Soc Nephrol.* 2016;27(7):2135-2147.

5. Holman RR, Paul SK, Bethel MA, Matthews DR, Neil HA. 10-year follow-up of intensive glucose control in type 2 diabetes. *N Engl J Med.* 2008;359(15):1577-1589.

6. Go AS, Chertow GM, Fan D, McCulloch CE, Hsu CY. Chronic kidney disease and the risks of death, cardiovascular events, and hospitalization. *N Engl J Med.* 2004;351(13):1296-1305.

7. Cea Soriano L, Johansson S, Stefansson B, Rodríguez LA. Cardiovascular events and all-cause mortality in a cohort of 57,946 patients with type 2 diabetes: associations with renal function and cardiovascular risk factors. *Cardiovasc Diabetol.* 2015;14:38.

8. Gaede P, Vedel P, Larsen N, Jensen GV, Parving HH, Pedersen O. Multifactorial intervention and cardiovascular disease in patients with type 2 diabetes. *N Engl J Med.* 2003;348(5):383-393.

9. Gaede P, Lund-Andersen H, Parving HH, Pedersen O. Effect of a multifactorial intervention on mortality in type 2 diabetes. *N Engl J Med.* 2008;358(6):580-591.

For peer review only - http://bmjopen.bmj.com/site/about/guidelines.xhtml

10. Wong ND, Patao C, Wong K, Malik S, Franklin SS, Iloeje U. Trends in control of cardiovascular risk factors among US adults with type 2 diabetes from 1999 to 2010: comparison by prevalent cardiovascular disease status. *Diab Vasc Dis Res.* 2013;10(6):505-513.

11. Wong ND, Zhao Y, Patel R, Patao C, Malik S, Bertoni AG, et al. Cardiovascular Risk Factor Targets and Cardiovascular Disease Event Risk in Diabetes: A Pooling Project of the Atherosclerosis Risk in Communities Study, Multi-Ethnic Study of Atherosclerosis, and Jackson Heart Study. *Diabetes Care.* 2016;39(5):668-676.

12. Vazquez-Benitez G, Desai JR, Xu S, Goodrich GK, Schroeder EB, Nichols GA, et al. Preventable major cardiovascular events associated with uncontrolled glucose, blood pressure, and lipids and active smoking in adults with diabetes with and without cardiovascular disease: a contemporary analysis. *Diabetes Care.* 2015;38(5):905-912.

13. Konstantinidis I, Nadkarni GN, Yacoub R, Saha A, Simoes P, Parikh CR, et al. Representation of Patients With Kidney Disease in Trials of Cardiovascular Interventions: An Updated Systematic Review. *JAMA Intern Med.* 2016;176(1):121-124.

For peer review only - http://bmjopen.bmj.com/site/about/guidelines.xhtml

14. Herrett E, Gallagher AM, Bhaskaran K, Forbes H, Mathur R, van Staa T, et al. Data Resource Profile: Clinical Practice Research Datalink (CPRD). *Int J Epidemiol.* 2015;44(3):827-836.

15. Nicholas J, Charlton J, Dregan A, Gulliford MC. Recent HbA1c values and mortality risk in type 2 diabetes. population-based case-control study. *PLoS One.* 2013;8(7):e68008.

16. Adamsson Eryd S, Gudbjörnsdottir S, Manhem K, Rosengren A, Svensson AM, Miftaraj M, et al. Blood pressure and complications in individuals with type 2 diabetes and no previous cardiovascular disease: national population based cohort study. *BMJ.* 2016;354:i4070.

17. Levey AS, Stevens LA, Schmid CH, Zhang YL, Castro AF 3rd, Feldman HI, et al. A new equation to estimate glomerular filtration rate. *Ann Intern Med.* 2009;150(9):604-612.

18. Kontopantelis E, Springate DA, Reeves D, Ashcroft DM, Rutter MK, Buchan I, et al. Glucose, blood pressure and cholesterol levels and their relationships to clinical outcomes in type 2 diabetes: a retrospective cohort study. *Diabetologia.* 2015;58(3):505-518.

19. Afghahi H, Svensson MK, Pirouzifard M, Eliasson B, Svensson AM. Blood

For peer review only - http://bmjopen.bmj.com/site/about/guidelines.xhtml

pressure level and risk of major cardiovascular events and all-cause of mortality in patients with type 2 diabetes and renal impairment: an observational study from the Swedish National Diabetes Register. *Diabetologia.* 2015;58(6):1203-1211.

20. Herrington W, Staplin N, Judge PK, Mafham M, Emberson J, Haynes R, et al. Evidence for Reverse Causality in the Association Between Blood Pressure and Cardiovascular Risk in Patients With Chronic Kidney Disease. *Hypertension.* 2017;69(2):314-322.

21. Weiner DE, Sarnak MJ. Managing dyslipidemia in chronic kidney disease. *J Gen Intern Med.* 2004;19(10):1045-1052.

22. Gulliford MC, Charlton J, Ashworth M, Rudd AG, Toschke AM; eCRT Research Team. Selection of medical diagnostic codes for analysis of electronic patient records. Application to stroke in a primary care database. *PLoS One.* 2009;4(9):e7168.

23. Bhattarai N, Charlton J, Rudisill C, Gulliford MC. Coding, recording and incidence of different forms of coronary heart disease in primary care. *PLoS One.* 2012;7(1):e29776.

24. Austin PC, Lee DS, Fine JP. Introduction to the Analysis of Survival Data in the Presence of Competing Risks. *Circulation.* 2016;133(6):601-609.

For peer review only

For peer review only - http://bmjopen.bmj.com/site/about/guidelines.xhtml

25. Gordon M, Lumley T. Advanced Forest Plot Using 'grid' Graphics. Vienna: The Comprehensive R Archive Network, 2016.

26. Anand SS, Islam S, Rosengren A, Franzosi MG, Steyn K, Yusufali AH, et al. Risk factors for myocardial infarction in women and men: insights from the INTERHEART study. *Eur Heart J.* 2008;29(7):932-940.

27. O'Donnell MJ, Xavier D, Liu L, Zhang H, Chin SL, Rao-Melacini P, et al. Risk factors for ischaemic and intracerebral haemorrhagic stroke in 22 countries (the INTERSTROKE study): a case-control study. *Lancet.* 2010;376(9735):112-123.

28. Chronic Kidney Disease Prognosis Consortium., Matsushita K, van der Velde M, Astor BC, et al. Association of estimated glomerular filtration rate and albuminuria with all-cause and cardiovascular mortality in general population cohorts: a collaborative meta-analysis. *Lancet.* 2010;375(9731):2073-2081.

29. Matsushita K, Coresh J, Sang Y, Chalmers J, Fox C, Guallar E, et al. Estimated glomerular filtration rate and albuminuria for prediction of cardiovascular outcomes: a collaborative meta-analysis of individual participant data. *Lancet Diabetes Endocrinol.* 2015;3(7):514-525.

30. Lee E, Oh HJ, Park JT, Han SH, Ryu DR, Kang SW, et al. The Incidence of Cardiovascular Events Is Comparable Between Normoalbuminuric and

For peer review only - http://bmjopen.bmj.com/site/about/guidelines.xhtml

Albuminuric Diabetic Patients With Chronic Kidney Disease. *Medicine (Baltimore)*. 2016;95(15):e3175.

For peer review only

For peer review only - http://bmjopen.bmj.com/site/about/guidelines.xhtml

Table 1. Baseline characteristics of the study cohort by CKD status

		CKD (N=11,431)	Non-CKD (N=36,429)	P value
Age (years)	Mean (SD)	71 (6)	62 (9)	<0.001
Gender	Male	5,481 (48)	22,006 (60)	<0.001
	Female	5,950 (52)	14,423 (40)	
eGFR (mL/min/1.73 m^2)	Mean (SD)	49 (9)	81 (13)	–
	15–29	558 (5)	–	
	30–44	2,655 (23)	–	
	45–59	8,218 (72)	–	
Smoking status	Non-smoker	5,426 (47)	16,511 (45)	<0.001
	Ex-smoker	4,327 (38)	12,217 (34)	
	Current smoker	1,678 (15)	7,701 (21)	
BMI (kg/m^2)	18.5–24.9	1,459 (13)	4,097 (11)	<0.001
	25.0–29.9	4,329 (38)	13,054 (36)	
	30.0–34.9	3,527 (31)	11,485 (32)	
	35.0–39.9	1,541 (13)	5,454 (15)	
	40.0–44.9	575 (5)	2,339 (6)	
Deprivation level (quintile)	1 (least deprived)	1,508 (13)	4,785 (13)	0.293
	2	2,331 (20)	7,300 (20)	
	3	2,374 (21)	7,640 (21)	
	4	2,637 (23)	8,172 (22)	
	5 (most deprived)	2,581 (23)	8,532 (23)	
Duration of diabetes (years)	1.0–4.9	5,208 (46)	22,527 (62)	<0.001
	5.0–9.9	2,954 (26)	8,356 (23)	
	≥10.0	3,269 (29)	5,546 (15)	
History of coronary heart disease and/or stroke		4,215 (37)	7,860 (22)	<0.001
HbA1c (mmol/mol/%)	42–47 (6.0–6.4)*	1,307 (11)	3,513 (10)	<0.001
	48–52 (6.5–6.9)	3,041 (27)	8,900 (24)	
	53–57 (7.0–7.4)	2,590 (23)	7,781 (21)	
	58–63 (7.5–7.9)	1,709 (15)	5,461 (15)	
	64–68 (8.0–8.4)	1,038 (9)	3,567 (10)	
	≥69 (≥8.5)	1,746 (15)	7,207 (20)	
Systolic blood pressure	120–129	1,777 (16)	7,203 (20)	<0.001

For peer review only - http://bmjopen.bmj.com/site/about/guidelines.xhtml

(mmHg)				
	130–139	3,508 (31)	12,121 (33)	
	140–149	3,387 (30)	10,242 (28)	
	≥150	2,759 (24)	6,863 (19)	
Diastolic blood pressure (mmHg)	60–79	7,238 (63)	16,803 (46)	<0.001
	80–89	3,599 (31)	15,816 (43)	
	≥90	594 (5)	3,810 (10)	
Total cholesterol (mmol/L)	3.0–3.9	3,782 (33)	10,960 (30)	<0.001
	4.0–4.9	5,220 (46)	16,387 (45)	
	≥5.0	2,429 (21)	9,082 (25)	
Medication	Antidiabetic drugs			<0.001
	Insulin (± non-insulin)	1,805 (16)	3,225 (9)	
	Non-insulin only	7,722 (68)	26,753 (73)	
	Antihypertensive drugs			<0.001
	Drugs on renin-angiotensin system (± others)	8,472 (74)	21,535 (59)	
	Other antihypertensive drugs only	1,610 (14)	4,751 (13)	
	Statins	9,004 (79)	27,011 (74)	<0.001
	Antiplatelet drugs	6,440 (56)	16,375 (45)	<0.001
Index year	2006	9,091 (80)	24,192 (66)	<0.001
	2007	1,008 (9)	3,741 (10)	
	2008	545 (5)	2,880 (8)	
	2009	432 (4)	2,677 (7)	
	2010	355 (3)	2,939 (8)	

BMI, body mass index; CKD, chronic kidney disease; eGFR, estimated glomerular filtration rate

* Participants with HbA1c <48 mmol/mol (<6.5%) were only included if they were prescribed antidiabetic drugs.

For peer review only

Table 2. Risk factors controlled according to chronic kidney disease and a history of cardiovascular diseases

	CKD			Non-CKD		
	Total (N=11,431)	No CVD (N=7,216)	CVD (N=4,215)	Total (N=36,429)	No CVD (N=28,569)	CVD (N=7,860)
Individual risk factor controlled						
HbA1c <53 mmol/mol (<7.0%)	4,348 (38)	2,767 (38)	1,581 (38)	12,413 (34)	9,603 (34)	2,810 (36)
Blood pressure <140 & <90 mmHg	5,224 (46)	3,147 (44)	2,077 (49)	18,655 (51)	14,438 (51)	4,217 (54)
Total cholesterol <5 mmol/L	9,002 (79)	5,512 (76)	3,490 (83)	27,347 (75)	20,826 (73)	6,521 (83)
No smoking	9,753 (85)	6,193 (86)	3,560 (84)	28,728 (79)	22,565 (79)	6,163 (78)
Number of risk factors controlled						
0	138 (1)	87 (1)	51 (1)	806 (2)	678 (2)	128 (2)
1	1,427 (12)	971 (13)	456 (11)	5,372 (15)	4,421 (15)	951 (12)
2	4,162 (36)	2,693 (37)	1,469 (35)	13,288 (36)	10,602 (37)	2,686 (34)
3	4,240 (37)	2,598 (36)	1,642 (39)	12,657 (35)	9,665 (34)	2,992 (38)
4	1,464 (13)	867 (12)	597 (14)	4,306 (12)	3,203 (11)	1,103 (14)

CKD, chronic kidney disease; CVD, (a history of) cardiovascular diseases

For peer review only - http://bmjopen.bmj.com/site/about/guidelines.xhtml

Figure legends

Figure 1. Relative hazards of the number of risk factors controlled for mortality and cardiovascular events in (a) participants with chronic kidney disease (CKD) and (b) participants with non-CKD. Hazard ratios (HRs) for all-cause mortality and subdistribution hazard ratios (SHRs) for cardiovascular mortality, coronary heart disease and stroke were adjusted for age, gender, CKD stage (for CKD cohort) body mass index, deprivation level, duration of diabetes, a history of cardiovascular diseases (for mortality evaluation), prescribing of antidiabetic, antihypertensive, statins and antiplatelet drugs, and index year.

Figure 2. Relative hazards of presence of chronic kidney disease for mortality and cardiovascular events compared with non-CKD as reference. Hazard ratios (HRs) for all-cause mortality and subdistribution hazard ratios (SHRs) for cardiovascular mortality, coronary heart disease and stroke were adjusted for age, gender, body mass index, deprivation level, duration of diabetes, a history of cardiovascular diseases (for mortality evaluation), prescribing of antidiabetic, antihypertensive, statins and antiplatelet drugs, and index year.

For peer review only - http://bmjopen.bmj.com/site/about/guidelines.xhtml

(a) CKD

Number of risk factors controlled	N	Number of events	Observation (1,000 py)	Rate (per 1,000 py)	Adjusted HR/SHR (95% CI)	P value
**All-cause mortality**						
4	1,464	388	8.1	48.1	0.59 (0.52 to 0.67)	<0.001
3	4,240	1,301	23.2	56.0	0.68 (0.62 to 0.75)	<0.001
2	4,162	1,393	22.6	61.5	0.76 (0.69 to 0.84)	<0.001
0+1	1,565	598	8.2	72.5	Reference	
**Cardiovascular mortality**						
4	1,464	236	8.1	29.3	0.58 (0.49 to 0.69)	<0.001
3	4,240	821	23.2	35.3	0.69 (0.61 to 0.78)	<0.001
2	4,162	893	22.6	39.4	0.78 (0.69 to 0.89)	<0.001
0+1	1,565	372	8.2	45.1	Reference	
**Coronary heart disease**						
4	867	143	4.5	32.0	0.73 (0.59 to 0.91)	0.004
3	2,598	510	13.3	38.4	0.85 (0.72 to 0.99)	0.037
2	2,693	558	13.4	41.6	0.88 (0.75 to 1.03)	0.106
0+1	1,058	232	5.1	45.5	Reference	
**Stroke**						
4	867	51	4.7	10.9	0.61 (0.43 to 0.86)	0.005
3	2,598	173	14.0	12.3	0.70 (0.54 to 0.89)	0.005
2	2,693	186	14.3	13.0	0.71 (0.55 to 0.90)	0.006
0+1	1,058	102	5.4	18.9	Reference	

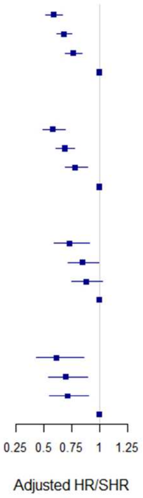

Figure 1a

284x173mm (96 x 96 DPI)

(b) Non-CKD

Number of risk factors controlled	N	Number of events	Observation (1,000 py)	Rate (per 1,000 py)	Adjusted HR/SHR (95% CI)	P value
**All-cause mortality**						
4	4,306	515	23.3	22.1	0.61 (0.54 to 0.68)	<0.001
3	12,657	1,670	69.7	23.9	0.68 (0.62 to 0.73)	<0.001
2	13,288	1,928	73.4	26.3	0.76 (0.70 to 0.83)	<0.001
0+1	6,178	935	33.3	28.1	Reference	
**Cardiovascular mortality**						
4	4,306	266	23.3	11.4	0.54 (0.46 to 0.62)	<0.001
3	12,657	912	69.	13.1	0.62 (0.56 to 0.69)	<0.001
2	13,288	1,015	73.4	13.8	0.67 (0.60 to 0.75)	<0.001
0+1	6,178	545	33.3	16.4	Reference	
**Coronary heart disease**						
4	3,203	305	16.6	18.4	0.54 (0.47 to 0.62)	<0.001
3	9,665	1,130	50.4	22.4	0.65 (0.59 to 0.71)	<0.001
2	10,602	1,343	54.8	24.5	0.70 (0.64 to 0.77)	<0.001
0+1	5,099	808	25.4	31.8	Reference	
**Stroke**						
4	3,203	63	17.2	3.7	0.37 (0.28 to 0.49)	<0.001
3	9,665	325	52.5	6.2	0.63 (0.53 to 0.74)	<0.001
2	10,602	424	57.2	7.4	0.74 (0.63 to 0.87)	<0.001
0+1	5,099	234	26.8	8.7	Reference	

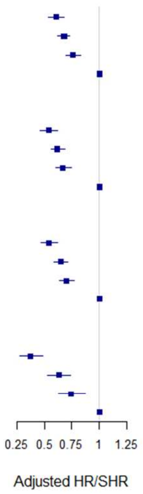

Figure 1b

287x172mm (96 x 96 DPI)

For peer review only

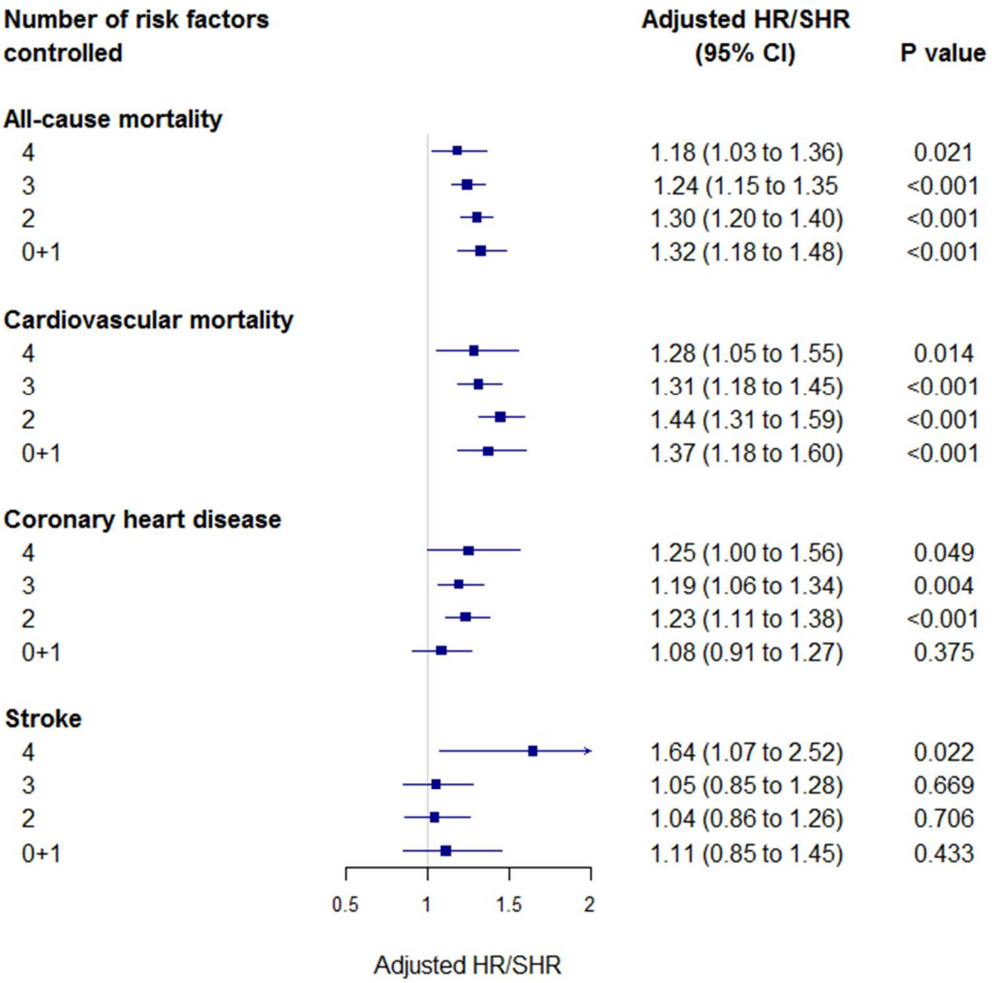

Figure 2

175x173mm (96 x 96 DPI)

SUPPLEMENTARY MATERIAL

Figure S1. Study cohort selection

Potentially eligible participants with diabetes between 2006 and 2010 (n=279,182)

- Participants who were diagnosed with type 1 or other specific types of diabetes
- Participants who were first diagnosed with diabetes before 30 years of age
- Participants who were first prescribed insulin within 180 days of the first diagnosis

Participants with type 2 diabetes (n=233,698)

Participants with a valid serum creatinine measurement between 2006 and 2010 (n=176,311)

Participants confirmed with prevalent diabetes using data within 12 months before the index date (n=127,792)

Participants aged 40 to 79 years at index date (n=108,502)

Participants with data at least 1 year before and after index date (n=106,799)

Participants with linked data for hospital care and death registration (n=66,100)

- Participants with a diagnosis of ESRD/RRT at baseline
- Participants with index eGFR <15 mL/min/1.73 m^2

For peer review only

For peer review only - http://bmjopen.bmj.com/site/about/guidelines.xhtml

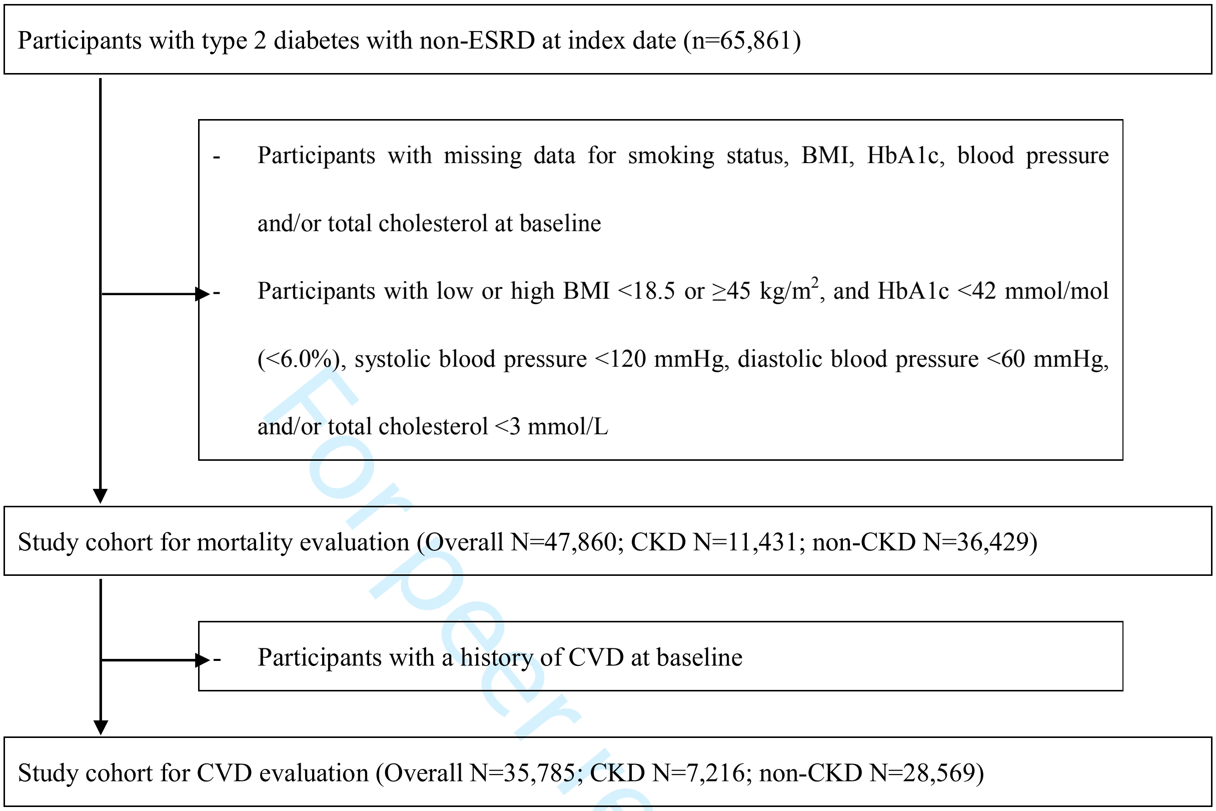

BMI, body mass index; CKD, chronic kidney disease; CVD, Cardiovascular diseases; eGFR, estimated glomerular filtration rate; ESRD, end-stage renal disease; RRT, renal replacement therapy

For peer review only

For peer review only - http://bmjopen.bmj.com/site/about/guidelines.xhtml

Table S1. Breakdown of risk factors controlled according to chronic kidney disease and a history of cardiovascular diseases

Number of risk factors controlled	HbA1c <53 mmol/mol (<7.0%)	Blood pressure <140/90 mmHg	Total cholesterol <5 mmol/L	No smoking	CKD Overall (N=11,431)	CKD No CVD (N=7,216)	CKD CVD (N=4,215)	Non-CKD Overall (N=36,429)	Non-CKD No CVD (N=28,569)	Non-CKD CVD (N=7,860)
0	–	–	–	–	138 (1)	87 (1)	51 (1)	806 (2)	678 (2)	128 (2)
1	Y	–	–	–	69 (0.6)	46 (0.6)	23 (0.6)	312 (1)	260 (0.9)	52 (0.7)
	–	Y	–	–	89 (0.8)	64 (0.9)	25 (0.6)	759 (2)	624 (2)	135 (2)
	–	–	Y	–	438 (4)	264 (4)	174 (4)	1,742 (5)	1,350 (5)	392 (5)
	–	–	–	Y	831 (7)	597 (8)	234 (6)	2,559 (7)	2,187 (8)	372 (5)
2	Y	Y	–	–	62 (0.5)	43 (0.6)	19 (0.5)	304 (0.8)	268 (0.9)	36 (0.5)
	Y	–	Y	–	245 (2)	152 (2)	93 (2)	794 (2)	590 (2)	204 (3)
	Y	–	–	Y	407 (4)	289 (4)	118 (3)	1,070 (3)	911 (3)	159 (2)
	–	Y	Y	–	390 (3)	215 (3)	175 (4)	1,911 (5)	1,446 (5)	465 (6)
	–	Y	–	Y	531 (5)	379 (5)	152 (4)	2,205 (6)	1,910 (7)	295 (4)
	–	–	Y	Y	2,527 (22)	1,615 (22)	912 (22)	7,004 (19)	5,477 (19)	1,527 (19)
3	Y	Y	Y	–	247 (2)	152 (2)	95 (2)	1,073 (3)	788 (3)	285 (4)
	Y	Y	–	Y	302 (3)	199 (3)	103 (2)	1,067 (3)	905 (3)	162 (2)
	Y	–	Y	Y	1,552 (14)	1,019 (14)	533 (13)	3,487 (10)	2,678 (9)	809 (10)
	–	Y	Y	Y	2,139 (19)	1,228 (17)	911 (22)	7,030 (19)	5,294 (19)	1,736 (22)
4	Y	Y	Y	Y	1,464 (13)	867 (12)	597 (14)	4,306 (12)	3,203 (11)	1,103 (14)

Y, meeting the criterion; –, not meeting the criterion

CKD, chronic kidney disease; CVD, (a history of) cardiovascular diseases

For peer review only

For peer review only - http://bmjopen.bmj.com/site/about/guidelines.xhtml

STROBE 2007 (v4) Statement—Checklist of items that should be included in reports of *cohort studies*

Section/Topic	Item #	Recommendation	Reported on page #
**Title and abstract**	1	(*a*) Indicate the study's design with a commonly used term in the title or the abstract	1,2
		(*b*) Provide in the abstract an informative and balanced summary of what was done and what was found	2,3
**Introduction**			
Background/rationale	2	Explain the scientific background and rationale for the investigation being reported	5,6
Objectives	3	State specific objectives, including any prespecified hypotheses	6
**Methods**			
Study design	4	Present key elements of study design early in the paper	6,7
Setting	5	Describe the setting, locations, and relevant dates, including periods of recruitment, exposure, follow-up, and data collection	6,7
Participants	6	(*a*) Give the eligibility criteria, and the sources and methods of selection of participants. Describe methods of follow-up	7,8,10
		(*b*) For matched studies, give matching criteria and number of exposed and unexposed	NA
Variables	7	Clearly define all outcomes, exposures, predictors, potential confounders, and effect modifiers. Give diagnostic criteria, if applicable	9
Data sources/ measurement	8*	For each variable of interest, give sources of data and details of methods of assessment (measurement). Describe comparability of assessment methods if there is more than one group	6-9
Bias	9	Describe any efforts to address potential sources of bias	10,11
Study size	10	Explain how the study size was arrived at	6-8
Quantitative variables	11	Explain how quantitative variables were handled in the analyses. If applicable, describe which groupings were chosen and why	11
Statistical methods	12	(*a*) Describe all statistical methods, including those used to control for confounding	11
		(*b*) Describe any methods used to examine subgroups and interactions	11
		(*c*) Explain how missing data were addressed	8
		(*d*) If applicable, explain how loss to follow-up was addressed	10
		(*e*) Describe any sensitivity analyses	NA
**Results**			

For peer review only

Participants	13*	(a) Report numbers of individuals at each stage of study—eg numbers potentially eligible, examined for eligibility, confirmed eligible, included in the study, completing follow-up, and analysed	12,Figure S1
		(b) Give reasons for non-participation at each stage	12,Figure S1
		(c) Consider use of a flow diagram	Figure S1
Descriptive data	14*	(a) Give characteristics of study participants (eg demographic, clinical, social) and information on exposures and potential confounders	12,Table 1
		(b) Indicate number of participants with missing data for each variable of interest	Figure S1
		(c) Summarise follow-up time (eg, average and total amount)	Figure 1
Outcome data	15*	Report numbers of outcome events or summary measures over time	Figure 1
Main results	16	(*a*) Give unadjusted estimates and, if applicable, confounder-adjusted estimates and their precision (eg, 95% confidence interval). Make clear which confounders were adjusted for and why they were included	12-14
		(*b*) Report category boundaries when continuous variables were categorized	9
		(*c*) If relevant, consider translating estimates of relative risk into absolute risk for a meaningful time period	NA
Other analyses	17	Report other analyses done—eg analyses of subgroups and interactions, and sensitivity analyses	NA
**Discussion**			
Key results	18	Summarise key results with reference to study objectives	14-15
**Limitations**			
Interpretation	20	Give a cautious overall interpretation of results considering objectives, limitations, multiplicity of analyses, results from similar studies, and other relevant evidence	15-17
Generalisability	21	Discuss the generalisability (external validity) of the study results	16,17
**Other information**			
Funding	22	Give the source of funding and the role of the funders for the present study and, if applicable, for the original study on which the present article is based	18

*Give information separately for cases and controls in case-control studies and, if applicable, for exposed and unexposed groups in cohort and cross-sectional studies.

Note: An Explanation and Elaboration article discusses each checklist item and gives methodological background and published examples of transparent reporting. The STROBE checklist is best used in conjunction with this article (freely available on the Web sites of PLoS Medicine at http://www.plosmedicine.org/, Annals of Internal Medicine at http://www.annals.org/, and Epidemiology at http://www.epidem.com/). Information on the STROBE Initiative is available at www.strobe-statement.org.

For peer review only

BMJ Open

Multiple risk factor control, mortality and cardiovascular events in type 2 diabetes and chronic kidney disease: a population-based cohort study

Journal:	*BMJ Open*
Manuscript ID	bmjopen-2017-019950.R1
Article Type:	Research
Date Submitted by the Author:	12-Jan-2018
Complete List of Authors:	Hamada, Shota; Institute for Health Economics and Policy, Research Department<br>Gulliford, Martin; King's College London, UK,
**Primary Subject Heading**:	Diabetes and endocrinology
Secondary Subject Heading:	General practice / Family practice
Keywords:	cardiovascular diseases, chronic kidney disease, mortality, type 2 diabetes

SCHOLARONE™
Manuscripts

For peer review only

Multiple risk factor control, mortality and cardiovascular events in type 2 diabetes and chronic kidney disease: a population-based cohort study

Shota Hamada[1,2], Martin C Gulliford[1,3]

1 Department of Primary Care and Public Health Sciences, King's College London, London, UK

2 Research Department, Institute for Health Economics and Policy, Association for Health Economics Research and Social Insurance and Welfare, Tokyo, Japan

3 National Institute for Health Research Biomedical Research Centre at Guy's and St Thomas' National Health Service Foundation Trust, London UK

Correspondence to: Shota Hamada

Research Department, Institute for Health Economics and Policy, Association for Health Economics Research and Social Insurance and Welfare

No.11 Toyo-kaiji Bldg, 1-5-11, Nishi-Shinbashi, Minato-ku, Tokyo, 105-0003 Japan

Tel: +81 3-3506-8529, Fax: +81 3-3506-8528, E-mail: shota.hamada@ihep.jp

For peer review only - http://bmjopen.bmj.com/site/about/guidelines.xhtml

Abstract

OBJECTIVES: This study aimed to evaluate the effectiveness of multiple risk factor control (MRFC) at reducing mortality and cardiovascular events in diabetes and chronic kidney disease (CKD) in clinical practice.

DESIGN: Population-based cohort study.

SETTING: Primary care database in the UK, linked with inpatient and mortality data.

PARTICIPANTS: Participants aged 40 to 79 years with type 2 diabetes and valid serum creatinine measurements, including 11,431 participants with CKD (eGFR 15–59 mL/min/1.73 m^2) and 36,429 participants with non-CKD (eGFR ≥60 mL/min/1.73 m^2).

EXPOSURES: MRFC consisted of four components: HbA1c <53 mmol/mol (<7.0%), blood pressure <140/90 mmHg, total cholesterol <5 mmol/L, and no smoking. The main exposure variable was the number of risk factors controlled at baseline.

OUTCOME MEASURES: All-cause and cardiovascular mortality in the overall participants. Cardiovascular events, including coronary heart disease and stroke, in participants limited to those without a history of cardiovascular diseases at baseline.

RESULTS: In participants with CKD, 37% or 13% met three or four MRFC criteria, respectively. Increasing numbers of risk factors controlled were associated with lower relative hazards for all outcomes studied compared with those meeting no or one

For peer review only - http://bmjopen.bmj.com/site/about/guidelines.xhtml

criterion. For participants with CKD meeting four criteria, the adjusted hazard ratio (HR) for all-cause mortality was 0.60 (95% confidence interval (CI) 0.53 to 0.69) and the adjusted subdistribution HR for cardiovascular mortality was 0.60 (0.50 to 0.70), considering a competing risk of non-cardiovascular death. Participants meeting four criteria also had lower relative hazards for coronary heart disease (adjusted subdistribution HR 0.73, 95% CI 0.59 to 0.91) and stroke (0.63, 0.45 to 0.89), considering death as a competing risk.

CONCLUSIONS: MRFC may lower the increased risks for mortality and cardiovascular events in people with diabetes and CKD. Further research is needed to evaluate appropriateness of MRFC according to individual participants' health status for improved management of cardiovascular risks in this population.

Strengths and limitations of this study

- This study included a large number of participants with type 2 diabetes and CKD sampled from a representative general population with about 6 years of follow-up, which enabled to determine the associations of cardiovascular risk factors with mortality and cardiovascular events.
- Linked data for hospital care and death registration with a primary care database

For peer review only - http://bmjopen.bmj.com/site/about/guidelines.xhtml

enhanced the validity of the study to evaluate mortality and cardiovascular events.

- We could not conclude that association represented causal relationships between MRFC and mortality and cardiovascular events in this non-randomised study.
- There is a possibility of confounding if healthier participants were managed more successfully and this resulted in being categorised as those with greater number of risk factors controlled.

For peer review only

For peer review only - http://bmjopen.bmj.com/site/about/guidelines.xhtml

Introduction

Diabetes and chronic kidney disease (CKD) are growing health problems worldwide, contributing to increased mortality [1]. Diabetes and CKD also impose a substantial economic burden on society, with particularly high costs relating to cardiovascular complications and renal replacement therapy [2,3]. The prevalence of CKD in patients with diabetes is between 4.2% and 17.9% (CKD stages 3 to 5) in European countries [4]. The leading cause of death in people with type 2 diabetes or CKD is cardiovascular disease rather than renal complications [5,6]. Prevention of cardiovascular events is a key focus in the management of patients with these conditions.

Multifactorial interventions to reduce cardiovascular risks were shown to be effective at reducing mortality and cardiovascular events in patients with type 2 diabetes and persistent microalbuminuria in the Steno-2 randomised trial [7,8]. This study provided a high level of evidence, but included a relatively small number of participants with diabetes who were managed in specialist centres. Recently, the implementation and effectiveness of this approach have been evaluated in patients with diabetes in clinical practice settings [9-11]. Epidemiological studies have demonstrated additional risks of CKD on mortality and cardiovascular diseases in people with diabetes [12], but

For peer review only - http://bmjopen.bmj.com/site/about/guidelines.xhtml

treatment approaches in this population have not been well studied. No studies focused on multiple risk factor control (MRFC) in patients with both diabetes and CKD in a wide clinical practice setting. Generally, patients with kidney disease have been underrepresented in cardiovascular clinical trials [13]. This population may have an altered risk-benefit profile, and extrapolation of data based on patients with normal kidney function into patients with CKD may be unreliable [13]. We aimed to conduct a pragmatic evaluation of the effectiveness of MRFC on mortality and cardiovascular events in participants with type 2 diabetes and CKD in a population-based cohort study.

Methods

Data sources

This study employed a linked dataset derived from the UK Clinical Practice Research Datalink (CPRD), the UK National Health Service Hospital Episodes Statistics (HES) inpatient data, and the UK Office for National Statistics (ONS) mortality data. The CPRD contains anonymised electronic health records from general practices across the UK [14]. The CPRD collects data for diagnoses and clinical assessment, prescriptions and laboratory test results, such as HbA1c and serum creatinine. The HES inpatient data were comprised of inpatient records from all National Health Service hospitals in

For peer review only - http://bmjopen.bmj.com/site/about/guidelines.xhtml

England. Information on the date of death and the causes of death were available in the ONS mortality data file. Multiple causes of death can be recorded in the mortality data. Diagnoses and clinical evaluation in the CPRD were coded with the Read codes, a hierarchical coding system used in primary care in the UK, whereas those in the HES and ONS were coded with the International Classification of Diseases, tenth revision (ICD-10). Linked data are available for general practices in England only and participants were limited to those with linked data for the HES and ONS available. The study was approved by the CPRD Independent Scientific Advisory Committee (ISAC Protocol 15_201R).

Study population

The scheme of the study cohort selection is presented in figure S1. We initially sampled participants who were diagnosed with type 2 diabetes from the CPRD [15]. Using the CPRD records, the date of the first valid serum creatinine value between 2006 and 2010 recorded more than one year after the first diagnosis of diabetes were defined as the index date. A similar approach was taken by Adamsson Eryd et al [16] to ensure that participants managed for diabetes had sufficient time available for recording of baseline values. To avoid misclassification of CKD status and stage, the index serum creatinine

For peer review only - http://bmjopen.bmj.com/site/about/guidelines.xhtml

values were validated by confirmation of subsequent values within 30% of the index values. We restricted the sample to participants aged 40 to 79 years at the index date with at least one year of follow-up data available (ie, participants who died in the first year of follow-up were excluded). Estimated glomerular filtration rate (eGFR) was calculated from a serum creatinine value, age, gender, and ethnicity, using the Chronic Kidney Disease Epidemiology Collaboration (CKD-EPI) equation [17]. Missing ethnicity was assumed as 'non-black' in the present study. Participants diagnosed with end-stage renal disease, those who had received renal replacement therapy, or those with index eGFR <15 mL/min/1.73 m^2 were excluded. We also excluded participants with missing data for smoking status, body mass index (BMI), HbA1c, blood pressure, or total cholesterol, or those with extreme BMI (<18.5 or ≥45 kg/m^2) at baseline. Since it has been reported that low values of cardiovascular risk factors were not always associated with better outcomes in observational studies [15,18,19], possibly due to reverse causation [20,21], participants with low HbA1c (<42 mmol/mol or <6.0%), blood pressure (systolic <120 or diastolic <60 mmHg), and total cholesterol (<3 mmol/L) were further excluded. Participants were categorised according to index eGFR into participants with CKD (<60 mL/min/1.73 m^2) and those with non-CKD (≥60 mL/min/1.73 m^2).

For peer review only - http://bmjopen.bmj.com/site/about/guidelines.xhtml

Multiple risk factor control

MRFC was defined in this study as consisting of four components: (1) HbA1c <53 mmol/mol (<7.0%), (2) blood pressure <140/90 mmHg (systolic <140 and diastolic <90 mmHg), (3) total cholesterol <5 mmol/L, and (4) no smoking (non- or ex-smokers). The means of HbA1c, blood pressure, and total cholesterol records within one year before the index date were evaluated. The number of the risk factors controlled from four criteria was treated as the exposure and included as a categorical variable in the analyses, with those meeting no or one criterion as a reference category.

Outcomes

Main outcomes of interest in this study included all-cause and cardiovascular mortality, fatal and non-fatal coronary heart disease (CHD) and stroke. The date of death and causes of death were determined using the ONS mortality data. Participants who died from cardiovascular causes were identified if people had any of the ICD-10 codes I00 to I99 as a cause of death. Similarly, participants who died from renal causes were identified by the ICD-10 codes N17 to N19. All of the CPRD, HES and ONS were used to ascertain fatal and non-fatal CHD and stroke. Read codes for CHD and stroke

For peer review only - http://bmjopen.bmj.com/site/about/guidelines.xhtml

reported previously [22,23] were updated for the present study. The ICD-10 codes for CHD and stroke were I20 to I25 and I60, I61, I63 and I64, respectively.

Analysis

Baseline characteristics of the study cohort were described according to CKD status. Time-to-event analyses were conducted to evaluate the associations of MRFC with mortality and cardiovascular events. To address the issue of reverse causation and to avoid misclassification of the outcomes from those which had existed at baseline, person-years for participants who experienced outcomes of interest in the first year of follow-up were excluded from analyses (figure S1). Cox proportional hazards models were used to evaluate the association of MRFC with all-cause mortality. Proportional hazards assumption was assessed by visual inspection of log-log plots, and no apparent violation was found. Competing risks regression with subdistribution hazard models were conducted for cardiovascular mortality and cardiovascular events, considering competing risks for non-cardiovascular and all-cause death, respectively [24]. Associations of MRFC with cardiovascular events were evaluated in participants without a known history of cardiovascular diseases at baseline (figure S1). Participants were followed from the index date until the earliest of the events of interest, the last date

For peer review only - http://bmjopen.bmj.com/site/about/guidelines.xhtml

of CPRD records, or 31 March 2015 for all-cause mortality evaluation. In the competing risks regression analyses for cardiovascular mortality and cardiovascular events, participants who experienced the corresponding competing events prior to the event of interest were also censored.

For peer review only

Main analyses were conducted by CKD status, adjusting for a range of baseline covariates, including age (continuous), gender (male or female), CKD stage (3a, 3b, and 4; for CKD cohort), BMI (18.5–24.9, 25.0–29.9, 30.0–34.9, 35.0–39.9, and 40.0–44.9 kg/m^2), deprivation level (quintile; 1, least deprived, to 5, most deprived), duration of diabetes (1.0–4.9 5.0–9.9, and 10+ years), proteinuria status, including microalbuminuria (yes, no, and a missing category), a history of cardiovascular diseases, including CHD and stroke (for mortality evaluation), and prescribing during six months prior to the index date of antidiabetic drugs (none, insulin with and without other antidiabetic drugs, and non-insulin drugs only), antihypertensive drugs (none, drugs acting on renin-angiotensin system with and without other antihypertensive drugs, and other classes of antihypertensive drugs only, including β-blockers, calcium channel blockers, and thiazide diuretics), statins and antiplatelet drugs, and index year (2006 to 2010). In addition, the associations of CKD with the outcomes were evaluated

For peer review only - http://bmjopen.bmj.com/site/about/guidelines.xhtml

according to the number of risk factors controlled, adjusting for the potential confounding factors described above.

In this paper, the results for participants with CKD were focused on, with the results for those with non-CKD shown for comparative purposes. The associations of each component of MRFC with the outcomes were also evaluated to aid interpretation of the study results. All analyses were performed using Stata version 14 (Stata Corp., College Station TX). The 'forestplot' package in R was used to present the results [25].

Results

Characteristics of the study population

Baseline characteristics of the study cohort are shown according to CKD status in table 1. Mean index eGFR was 49 mL/min/1.73 m^2 for participants with CKD and 81 mL/min/1.73 m^2 for those with non-CKD. Participants with CKD were older (71 *vs* 62 years), included more women (52% *vs* 40%), had a longer duration of diabetes, and were more likely to have a history of cardiovascular diseases (37% *vs* 22%). A higher frequency of proteinuria was recorded in participants with CKD (18% *vs* 12% among participants with records of proteinuria status). HbA1c and total cholesterol were

For peer review only - http://bmjopen.bmj.com/site/about/guidelines.xhtml

slightly lower in participants with CKD. Although diastolic blood pressure was lower in participants with CKD, systolic blood pressure was higher despite more people under antihypertensive medications. Participants with CKD were prescribed insulin, drugs on renin-angiotensin systems, statins, and antiplatelet drugs more frequently.

Implementation of MRFC

The number of risk factors controlled from four components of MRFC are shown in table 2. More detailed results of which of the components were controlled are available in table S1. Higher rates of control for HbA1c, total cholesterol, and smoking status were observed in participants with CKD compared with those with non-CKD. However, blood pressure was less likely managed in participants with CKD (46% *vs* 51%). There were some differences in management status according to a history of cardiovascular diseases: in participants with CKD, higher rates of control of blood pressure (49% *vs* 44%) and total cholesterol (83% *vs* 76%) in participants with a history of cardiovascular diseases compared with those without. Participants meeting three or four criteria accounted for 37% or 13% in participants with CKD.

Effectiveness of MRFC

For peer review only

Absolute risks for mortality and cardiovascular diseases and adjusted relative hazards of the number of risk factors controlled for the outcomes are shown in figure 1. Increasing numbers of risk factors controlled were associated with lower relative hazards for all outcomes studied relative to participants meeting no or one criterion. For participants with CKD meeting four MRFC criteria, the adjusted hazard ratio (HR) for all-cause mortality was 0.60 (95% CI 0.53 to 0.69), and adjusted subdistribution HR for cardiovascular mortality was 0.60 (0.50 to 0.70). Participants meeting four criteria also had lower relative risks for CHD (adjusted subdistribution HR 0.73, 95% CI 0.59 to 0.91) and stroke (0.63, 0.45 to 0.89) in participants with CKD. In participants with non-CKD, increasing numbers of risk factors controlled were also associated with lower risks for all-cause and cardiovascular mortality, CHD, and stroke. As shown in figure S2, the strengths of associations of each component of MRFC with mortality and cardiovascular diseases were different; for example, the greatest associations of no smoking with all-cause and cardiovascular mortality were observed in participants with and without CKD.

Comparisons between CKD and non-CKD

Unadjusted absolute risks for mortality and cardiovascular diseases were higher in

For peer review only - http://bmjopen.bmj.com/site/about/guidelines.xhtml

participants with CKD by 1.4- to 2.9-fold compared with those with non-CKD at the same MRFC category (figure 1). More participants with CKD died from cardiovascular causes compared with those without (63% *vs* 54%, P<0.001). More participants with CKD died from renal causes (n=631 or 5% *vs* n=326 or 0.9%, P<0.001), but the proportions were much smaller than cardiovascular causes of death. Relative hazards of CKD for the outcomes are shown in figure 2. After adjustment with possible confounding factors, comorbid CKD remained to be associated with greater risks for all-cause mortality (adjusted HR, 1.16 to 1.30) and cardiovascular mortality (adjusted subdistribution HR, 1.25 to 1.41). In participants meeting two or more criteria, comorbid CKD was associated with a higher risk for CHD (1.18 to 1.25). The associations of comorbid CKD with stroke was observed in participants meeting four criteria only (1.64).

Discussion

In this population-based cohort study of participants with type 2 diabetes and CKD stages 3 to 4, MRFC was associated with lower relative risks for mortality (N>11,000) and cardiovascular diseases (N>7,000). We also confirmed that CKD was associated with increased risks for mortality and cardiovascular events. Higher absolute risks for

For peer review only

For peer review only - http://bmjopen.bmj.com/site/about/guidelines.xhtml

mortality and cardiovascular events and great relative risk reduction associated with MRFC suggest that the MRFC strategy may be one of the main approaches to potentially reducing the burden of diabetes and CKD.

This study evaluated the effectiveness of MRFC in patients with type 2 diabetes according to presence or absence of CKD in clinical practice. So far, the associations of MRFC with lower risks for mortality and cardiovascular events have been shown in people with diabetes, not focusing on CKD status. Participants with controlled three risk factors of HbA1c, blood pressure, and LDL cholesterol had 62% and 60% risk reduction for cardiovascular events and CHD, respectively, in patients with diabetes without known cardiovascular diseases [10]. The associations of uncontrolled HbA1c, blood pressure, LDL cholesterol, and smoking with mortality and cardiovascular events were individually evaluated in a large population-based study with >850,000 participants with diabetes [11]. The study cohort included 35.5% of participants with CKD in those with cardiovascular diseases and 21.8% in those without, and CKD was included in the analyses for adjustment. This study suggested that uncontrolled risk factors attributed to about 1 in 3 major cardiovascular events and fewer 1 in 10 deaths.

For peer review only - http://bmjopen.bmj.com/site/about/guidelines.xhtml

The strength of this study was the inclusion of a large size of >11,000 participants with diabetes and CKD with an observation of >62,000 person-years. In addition to the large sample size and long-term follow-up, representativeness from general population and data quality are also advantages of the CPRD [14], which should remain even if linked data for HES and ONS are only available for England practices. Instead, linked data for hospital care and death registration substantially enhanced the validity of the study to evaluate mortality and cardiovascular events.

There are also some limitations in this study. First, despite our focus on the number of MRFC, the impacts of each component of MRFC on mortality and cardiovascular events were different. Different cut-off points for HbA1c, blood pressure, and total cholesterol may bring different results. Next, we could not conclude that associations represented causal relationships between MRFC and mortality and cardiovascular events in this non-randomised study. There is a possibility of confounding if healthier participants were managed more successfully and this resulted in being categorised as those with greater number of risk factors controlled. For example, stringent management of HbA1c might not be targeted for vulnerable participants due to concerns for greater risk of hypoglycaemia, a form of confounding by contra-indication. We

For peer review only - http://bmjopen.bmj.com/site/about/guidelines.xhtml

cannot exclude the possibility of residual confounding despite adjustment with a range of covariates in the analyses, including physical activity and alcohol intake [26,27]. Then, measurement and assay methods for HbA1c, blood pressure, cholesterol and serum creatinine might not have been standardised among general practices or laboratories. As well as missing data on ethnicity and fluctuations in serum creatinine values, these methodological limitations might influence the determination of CKD status or staging. Finally, although we used one of the largest primary care electronic health records database, it seemed to be insufficient to separately evaluate MRFC for participants with different stages of CKD. Further research is needed to focus on patients with more advanced CKD who may have altered risk-benefit profile compared with patients with less impaired renal function.

In summary, based on the population-based cohort study of routine clinical practices, MRFC may lower the increased risks for mortality and cardiovascular events in people with diabetes and CKD. Further research is needed to evaluate appropriateness of MRFC according to individual participants' health status for improved management of cardiovascular risks in this population.

For peer review only - http://bmjopen.bmj.com/site/about/guidelines.xhtml

Acknowledgments

SH was supported by the Uehara Memorial Foundation Postdoctoral Fellowship, Tokyo, Japan. MCG was supported by the National Institute for Health Research (NIHR) Biomedical Research Centre at Guy's and St Thomas' NHS Foundation Trust and King's College London. This study is based on data from the CPRD obtained under license from the UK Medicines and Healthcare products Regulatory Agency. However, the interpretation and conclusions contained in this report are those of the authors alone and not necessarily those of the National Health Service, the NIHR or the Department of Health.

Contributors: Both authors contributed to conception and study design of the study, data acquisition, statistical analysis, and interpretation. SH drafted the manuscript and MCG revised it critically for important intellectual content.

Funding: This research received no specific grant from any funding agency.

Competing interests: None declared.

Provenance and peer review: Not commissioned; externally peer reviewed.

Data sharing statement: No additional data are available.

For peer review only - http://bmjopen.bmj.com/site/about/guidelines.xhtml

References

1. GBD 2013 Mortality and Causes of Death Collaborators. Global, regional, and national age-sex specific all-cause and cause-specific mortality for 240 causes of death, 1990-2013: a systematic analysis for the Global Burden of Disease Study 2013. *Lancet.* 2015;385(9963):117-171.

2. Seuring T, Archangelidi O, Suhrcke M. The Economic Costs of Type 2 Diabetes: A Global Systematic Review. *Pharmacoeconomics.* 2015;33(8):811-831.

3. Kerr M, Bray B, Medcalf J, O'Donoghue DJ, Matthews B. Estimating the financial cost of chronic kidney disease to the NHS in England. *Nephrol Dial Transplant.* 2012;27 Suppl 3:iii73-80.

4. Brück K, Stel VS, Gambaro G, Hallan S, Völzke H, Ärnlöv J, et al. CKD Prevalence Varies across the European General Population. *J Am Soc Nephrol.* 2016;27(7):2135-2147.

5. Holman RR, Paul SK, Bethel MA, Matthews DR, Neil HA. 10-year follow-up of intensive glucose control in type 2 diabetes. *N Engl J Med.* 2008;359(15):1577-1589.

6. Go AS, Chertow GM, Fan D, McCulloch CE, Hsu CY. Chronic kidney disease and the risks of death, cardiovascular events, and hospitalization. *N Engl J Med.*

For peer review only - http://bmjopen.bmj.com/site/about/guidelines.xhtml

2004;351(13):1296-1305.

7. Gaede P, Vedel P, Larsen N, Jensen GV, Parving HH, Pedersen O. Multifactorial intervention and cardiovascular disease in patients with type 2 diabetes. *N Engl J Med.* 2003;348(5):383-393.

8. Gaede P, Lund-Andersen H, Parving HH, Pedersen O. Effect of a multifactorial intervention on mortality in type 2 diabetes. *N Engl J Med.* 2008;358(6):580-591.

9. Wong ND, Patao C, Wong K, Malik S, Franklin SS, Iloeje U. Trends in control of cardiovascular risk factors among US adults with type 2 diabetes from 1999 to 2010: comparison by prevalent cardiovascular disease status. *Diab Vasc Dis Res.* 2013;10(6):505-513.

10. Wong ND, Zhao Y, Patel R, Patao C, Malik S, Bertoni AG, et al. Cardiovascular Risk Factor Targets and Cardiovascular Disease Event Risk in Diabetes: A Pooling Project of the Atherosclerosis Risk in Communities Study, Multi-Ethnic Study of Atherosclerosis, and Jackson Heart Study. *Diabetes Care.* 2016;39(5):668-676.

11. Vazquez-Benitez G, Desai JR, Xu S, Goodrich GK, Schroeder EB, Nichols GA, et al. Preventable major cardiovascular events associated with uncontrolled glucose, blood pressure, and lipids and active smoking in adults with diabetes with and

For peer review only - http://bmjopen.bmj.com/site/about/guidelines.xhtml

without cardiovascular disease: a contemporary analysis. *Diabetes Care.* 2015;38(5):905-912.

12. Cea Soriano L, Johansson S, Stefansson B, Rodríguez LA. Cardiovascular events and all-cause mortality in a cohort of 57,946 patients with type 2 diabetes: associations with renal function and cardiovascular risk factors. *Cardiovasc Diabetol.* 2015;14:38.

13. Konstantinidis I, Nadkarni GN, Yacoub R, Saha A, Simoes P, Parikh CR, et al. Representation of Patients With Kidney Disease in Trials of Cardiovascular Interventions: An Updated Systematic Review. *JAMA Intern Med.* 2016;176(1):121-124.

14. Herrett E, Gallagher AM, Bhaskaran K, Forbes H, Mathur R, van Staa T, et al. Data Resource Profile: Clinical Practice Research Datalink (CPRD). *Int J Epidemiol.* 2015;44(3):827-836.

15. Nicholas J, Charlton J, Dregan A, Gulliford MC. Recent HbA1c values and mortality risk in type 2 diabetes. population-based case-control study. *PLoS One.* 2013;8(7):e68008.

16. Adamsson Eryd S, Gudbjörnsdottir S, Manhem K, Rosengren A, Svensson AM, Miftaraj M, et al. Blood pressure and complications in individuals with type 2

For peer review only

For peer review only - http://bmjopen.bmj.com/site/about/guidelines.xhtml

diabetes and no previous cardiovascular disease: national population based cohort study. *BMJ.* 2016;354:i4070.

17. Levey AS, Stevens LA, Schmid CH, Zhang YL, Castro AF 3rd, Feldman HI, et al. A new equation to estimate glomerular filtration rate. *Ann Intern Med.* 2009;150(9):604-612.

18. Kontopantelis E, Springate DA, Reeves D, Ashcroft DM, Rutter MK, Buchan I, et al. Glucose, blood pressure and cholesterol levels and their relationships to clinical outcomes in type 2 diabetes: a retrospective cohort study. *Diabetologia.* 2015;58(3):505-518.

19. Afghahi H, Svensson MK, Pirouzifard M, Eliasson B, Svensson AM. Blood pressure level and risk of major cardiovascular events and all-cause of mortality in patients with type 2 diabetes and renal impairment: an observational study from the Swedish National Diabetes Register. *Diabetologia.* 2015;58(6):1203-1211.

20. Herrington W, Staplin N, Judge PK, Mafham M, Emberson J, Haynes R, et al. Evidence for Reverse Causality in the Association Between Blood Pressure and Cardiovascular Risk in Patients With Chronic Kidney Disease. *Hypertension.* 2017;69(2):314-322.

21. Weiner DE, Sarnak MJ. Managing dyslipidemia in chronic kidney disease. *J Gen*

For peer review only

For peer review only - http://bmjopen.bmj.com/site/about/guidelines.xhtml

Intern Med. 2004;19(10):1045-1052.

22. Gulliford MC, Charlton J, Ashworth M, Rudd AG, Toschke AM; eCRT Research Team. Selection of medical diagnostic codes for analysis of electronic patient records. Application to stroke in a primary care database. *PLoS One.* 2009;4(9):e7168.

23. Bhattarai N, Charlton J, Rudisill C, Gulliford MC. Coding, recording and incidence of different forms of coronary heart disease in primary care. *PLoS One.* 2012;7(1):e29776.

24. Austin PC, Lee DS, Fine JP. Introduction to the Analysis of Survival Data in the Presence of Competing Risks. *Circulation.* 2016;133(6):601-609.

25. Gordon M, Lumley T. Advanced Forest Plot Using 'grid' Graphics. Vienna: The Comprehensive R Archive Network, 2016.

26. Anand SS, Islam S, Rosengren A, Franzosi MG, Steyn K, Yusufali AH, et al. Risk factors for myocardial infarction in women and men: insights from the INTERHEART study. *Eur Heart J.* 2008;29(7):932-940.

27. O'Donnell MJ, Xavier D, Liu L, Zhang H, Chin SL, Rao-Melacini P, et al. Risk factors for ischaemic and intracerebral haemorrhagic stroke in 22 countries (the INTERSTROKE study): a case-control study. *Lancet.* 2010;376(9735):112-123.

For peer review only - http://bmjopen.bmj.com/site/about/guidelines.xhtml

For peer review only

For peer review only - http://bmjopen.bmj.com/site/about/guidelines.xhtml

Table 1. Baseline characteristics of the study cohort by CKD status

		CKD (N=11,431)	Non-CKD (N=36,429)	P value
Age (years)	Mean (SD)	71 (6)	62 (9)	<0.001
Gender	Male	5,481 (48)	22,006 (60)	<0.001
	Female	5,950 (52)	14,423 (40)	
eGFR (mL/min/1.73 m^2)	Mean (SD)	49 (9)	81 (13)	–
	15–29	558 (5)	–	
	30–44	2,655 (23)	–	
	45–59	8,218 (72)	–	
Smoking status	Non-smoker	5,426 (47)	16,511 (45)	<0.001
	Ex-smoker	4,327 (38)	12,217 (34)	
	Current smoker	1,678 (15)	7,701 (21)	
BMI (kg/m^2)	18.5–24.9	1,459 (13)	4,097 (11)	<0.001
	25.0–29.9	4,329 (38)	13,054 (36)	
	30.0–34.9	3,527 (31)	11,485 (32)	
	35.0–39.9	1,541 (13)	5,454 (15)	
	40.0–44.9	575 (5)	2,339 (6)	
Deprivation level (quintile)	1 (least deprived)	1,508 (13)	4,785 (13)	0.293
	2	2,331 (20)	7,300 (20)	
	3	2,374 (21)	7,640 (21)	
	4	2,637 (23)	8,172 (22)	
	5 (most deprived)	2,581 (23)	8,532 (23)	
Duration of diabetes (years)	1.0–4.9	5,208 (46)	22,527 (62)	<0.001
	5.0–9.9	2,954 (26)	8,356 (23)	
	≥10.0	3,269 (29)	5,546 (15)	
Proteinuria	Yes	1,714 (15)	3,279 (9)	<0.001
	No	7,666 (67)	24,110 (66)	
	Missing	2,051 (18)	9,040 (25)	
History of coronary heart disease and/or stroke		4,215 (37)	7,860 (22)	<0.001
HbA1c (mmol/mol/%)	42–47 (6.0–6.4)*	1,307 (11)	3,513 (10)	<0.001
	48–52 (6.5–6.9)	3,041 (27)	8,900 (24)	
	53–57 (7.0–7.4)	2,590 (23)	7,781 (21)	
	58–63 (7.5–7.9)	1,709 (15)	5,461 (15)	

For peer review only - http://bmjopen.bmj.com/site/about/guidelines.xhtml

For peer review only

	64–68 (8.0–8.4)	1,038 (9)	3,567 (10)	
	≥69 (≥8.5)	1,746 (15)	7,207 (20)	
Systolic blood pressure (mmHg)	120–129	1,777 (16)	7,203 (20)	<0.001
	130–139	3,508 (31)	12,121 (33)	
	140–149	3,387 (30)	10,242 (28)	
	≥150	2,759 (24)	6,863 (19)	
Diastolic blood pressure (mmHg)	60–79	7,238 (63)	16,803 (46)	<0.001
	80–89	3,599 (31)	15,816 (43)	
	≥90	594 (5)	3,810 (10)	
Total cholesterol (mmol/L)	3.0–3.9	3,782 (33)	10,960 (30)	<0.001
	4.0–4.9	5,220 (46)	16,387 (45)	
	≥5.0	2,429 (21)	9,082 (25)	
Medication	Antidiabetic drugs			<0.001
	Insulin (± non-insulin)	1,805 (16)	3,225 (9)	
	Non-insulin only	7,722 (68)	26,753 (73)	
	Antihypertensive drugs			<0.001
	Drugs on renin-angiotensin system (± others)	8,472 (74)	21,535 (59)	
	Other antihypertensive drugs only	1,610 (14)	4,751 (13)	
	Statins	9,004 (79)	27,011 (74)	<0.001
	Antiplatelet drugs	6,440 (56)	16,375 (45)	<0.001
Index year	2006	9,091 (80)	24,192 (66)	<0.001
	2007	1,008 (9)	3,741 (10)	
	2008	545 (5)	2,880 (8)	
	2009	432 (4)	2,677 (7)	
	2010	355 (3)	2,939 (8)	

BMI, body mass index; CKD, chronic kidney disease; eGFR, estimated glomerular filtration rate

Frequencies (percentages) are shown otherwise specified.

* Participants with HbA1c <48 mmol/mol (<6.5%) were only included if they were prescribed antidiabetic drugs.

For peer review only - http://bmjopen.bmj.com/site/about/guidelines.xhtml

For peer review only

Table 2. Risk factors controlled according to chronic kidney disease and a history of cardiovascular diseases

	CKD			Non-CKD		
	Total (N=11,431)	No CVD (N=7,216)	CVD (N=4,215)	Total (N=36,429)	No CVD (N=28,569)	CVD (N=7,860)
Individual risk factor controlled						
HbA1c <53 mmol/mol (<7.0%)	4,348 (38)	2,767 (38)	1,581 (38)	12,413 (34)	9,603 (34)	2,810 (36)
Blood pressure <140 & <90 mmHg	5,224 (46)	3,147 (44)	2,077 (49)	18,655 (51)	14,438 (51)	4,217 (54)
Total cholesterol <5 mmol/L	9,002 (79)	5,512 (76)	3,490 (83)	27,347 (75)	20,826 (73)	6,521 (83)
No smoking	9,753 (85)	6,193 (86)	3,560 (84)	28,728 (79)	22,565 (79)	6,163 (78)
Number of risk factors controlled						
0	138 (1)	87 (1)	51 (1)	806 (2)	678 (2)	128 (2)
1	1,427 (12)	971 (13)	456 (11)	5,372 (15)	4,421 (15)	951 (12)
2	4,162 (36)	2,693 (37)	1,469 (35)	13,288 (36)	10,602 (37)	2,686 (34)
3	4,240 (37)	2,598 (36)	1,642 (39)	12,657 (35)	9,665 (34)	2,992 (38)
4	1,464 (13)	867 (12)	597 (14)	4,306 (12)	3,203 (11)	1,103 (14)

CKD, chronic kidney disease; CVD, (a history of) cardiovascular diseases

Frequencies (percentages) are shown.

For peer review only

Figure legends

Figure 1. Relative hazards of the number of risk factors controlled for mortality and cardiovascular events in (a) participants with chronic kidney disease (CKD) and (b) participants with non-CKD. Hazard ratios (HRs) for all-cause mortality and subdistribution hazard ratios (SHRs) for cardiovascular mortality, coronary heart disease, and stroke were adjusted for age, gender, CKD stage (for CKD cohort), body mass index, deprivation level, duration of diabetes, proteinuria status, a history of cardiovascular diseases (for mortality evaluation), prescribing of antidiabetic, antihypertensive, statins and antiplatelet drugs, and index year.

Figure 2. Relative hazards of presence of chronic kidney disease for mortality and cardiovascular events compared with non-CKD as reference. Hazard ratios (HRs) for all-cause mortality and subdistribution hazard ratios (SHRs) for cardiovascular mortality, coronary heart disease, and stroke were adjusted for age, gender, body mass index, deprivation level, duration of diabetes, proteinuria status, a history of cardiovascular diseases (for mortality evaluation), prescribing of antidiabetic, antihypertensive, statins and antiplatelet drugs, and index year.

For peer review only - http://bmjopen.bmj.com/site/about/guidelines.xhtml

(a) CKD

Number of risk factors controlled	N	Number of events	Observation (1,000 py)	Rate (per 1,000 py)	Adjusted HR/SHR (95% CI)	P value
**[All participants]**						
**All-cause mortality**						
4	1,464	388	8.1	48.1	0.60 (0.53 to 0.69)	<0.001
3	4,240	1,301	23.2	56.0	0.70 (0.63 to 0.77)	<0.001
2	4,162	1,393	22.6	61.5	0.77 (0.70 to 0.85)	<0.001
0+1	1,565	598	8.2	72.5	Reference	
**Cardiovascular mortality**						
4	1,464	236	8.1	29.3	0.60 (0.50 to 0.70)	<0.001
3	4,240	821	23.2	35.3	0.71 (0.62 to 0.80)	<0.001
2	4,162	893	22.6	39.4	0.79 (0.70 to 0.90)	<0.001
0+1	1,565	372	8.2	45.1	Reference	
**[Participants without previous CHD/stroke]**						
**Coronary heart disease**						
4	851	143	4.5	32.0	0.73 (0.59 to 0.91)	0.004
3	2,537	510	13.3	38.4	0.86 (0.73 to 1.00)	0.053
2	2,635	558	13.4	41.6	0.88 (0.75 to 1.03)	0.114
0+1	1,025	232	5.1	45.5	Reference	
**Stroke**						
4	865	51	4.7	10.9	0.63 (0.45 to 0.89)	0.009
3	2,580	173	14.0	12.3	0.72 (0.56 to 0.92)	0.010
2	2,670	186	14.3	13.0	0.72 (0.56 to 0.93)	0.010
0+1	1,044	102	5.4	18.9	Reference	

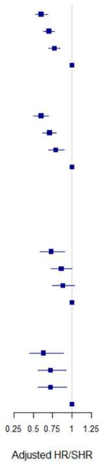

(b) Non-CKD

Number of risk factors controlled	N	Number of events	Observation (1,000 py)	Rate (per 1,000 py)	Adjusted HR/SHR (95% CI)	P value
**[All participants]**						
**All-cause mortality**						
4	4,306	515	23.3	22.1	0.62 (0.55 to 0.69)	<0.001
3	12,657	1,670	69.7	23.9	0.69 (0.63 to 0.74)	<0.001
2	13,288	1,928	73.4	26.3	0.77 (0.71 to 0.83)	<0.001
0+1	6,178	935	33.3	28.1	Reference	
**Cardiovascular mortality**						
4	4,306	266	23.3	11.4	0.55 (0.47 to 0.64)	<0.001
3	12,657	912	69.7	13.1	0.63 (0.57 to 0.70)	<0.001
2	13,288	1,015	73.4	13.8	0.68 (0.61 to 0.75)	<0.001
0+1	6,178	545	33.3	16.4	Reference	
**[Participants without previous CHD/stroke]**						
**Coronary heart disease**						
4	3,164	305	16.6	18.4	0.54 (0.47 to 0.62)	<0.001
3	9,528	1,130	50.4	22.4	0.65 (0.59 to 0.71)	<0.001
2	10,446	1,343	54.8	24.5	0.70 (0.64 to 0.77)	<0.001
0+1	5,010	808	25.4	31.8	Reference	
**Stroke**						
4	3,190	63	17.2	3.7	0.37 (0.28 to 0.50)	<0.001
3	9,628	325	52.5	6.2	0.63 (0.53 to 0.75)	<0.001
2	10,565	424	57.2	7.4	0.75 (0.63 to 0.88)	<0.001
0+1	5,079	234	26.8	8.7	Reference	

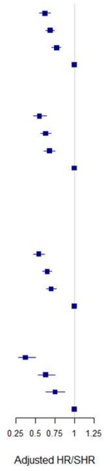

figure1

105x156mm (300 x 300 DPI)

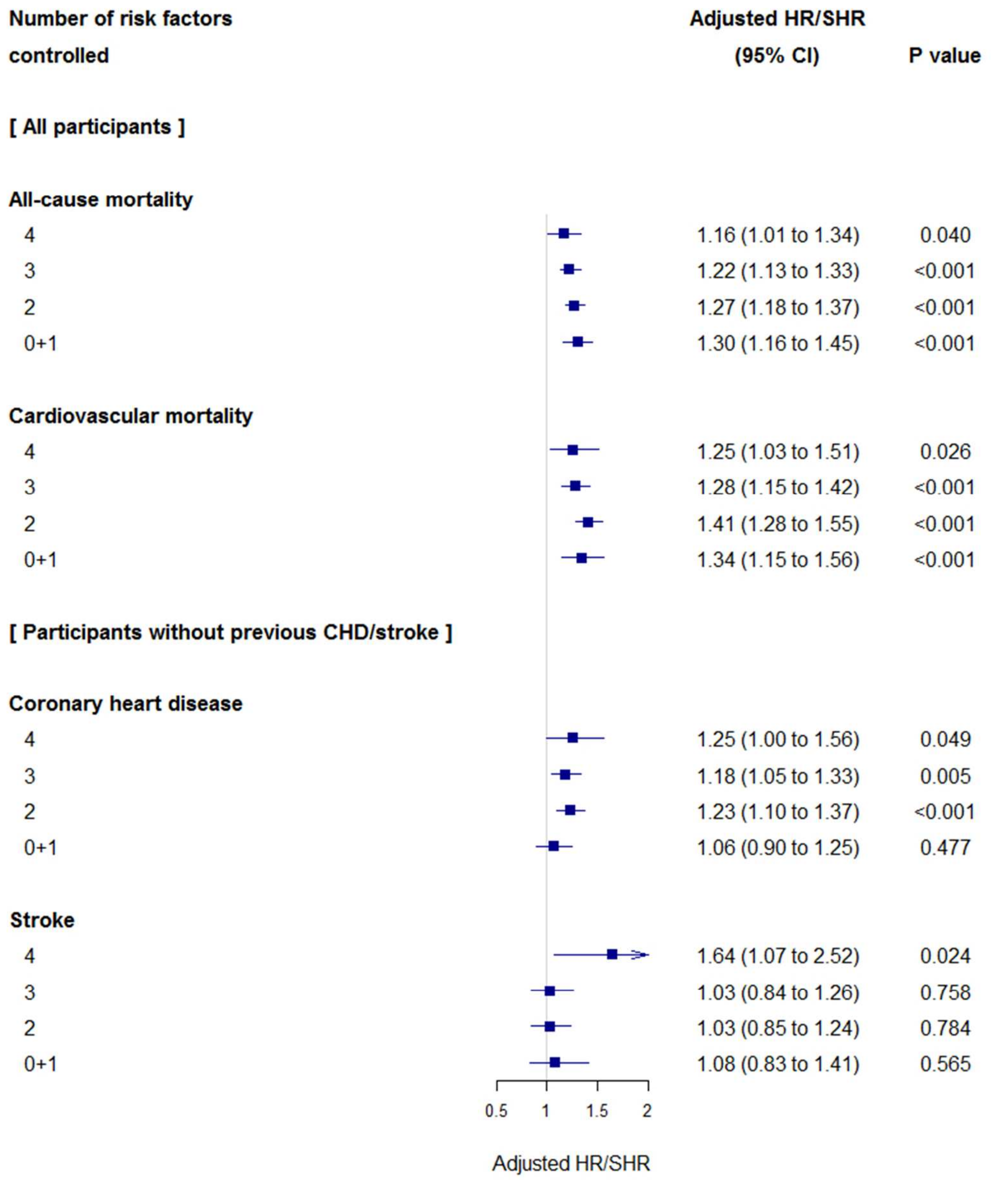

figure2

66x78mm (300 x 300 DPI)

SUPPLEMENTARY MATERIAL

Figure S1. Study cohort selection

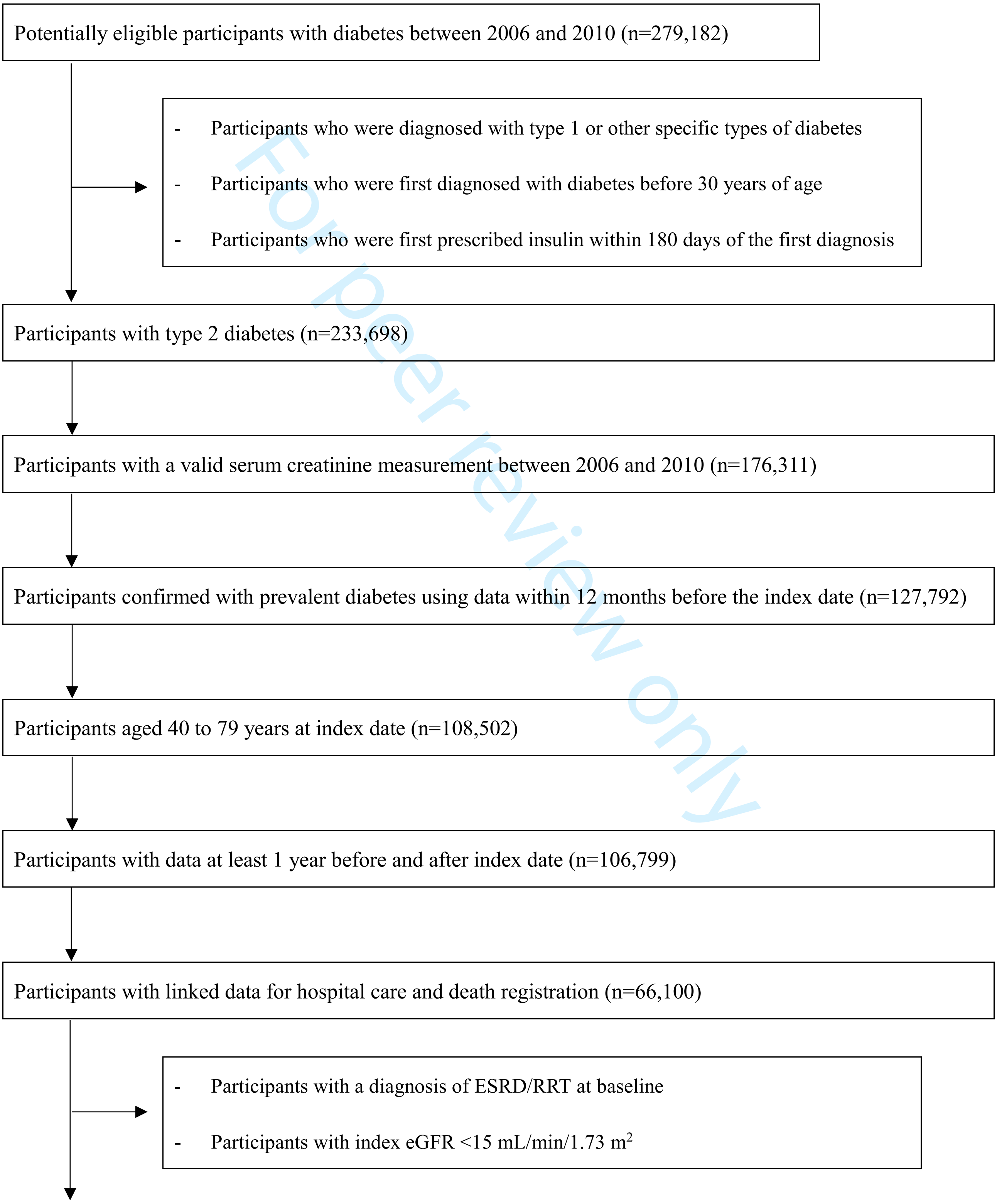

For peer review only - http://bmjopen.bmj.com/site/about/guidelines.xhtml

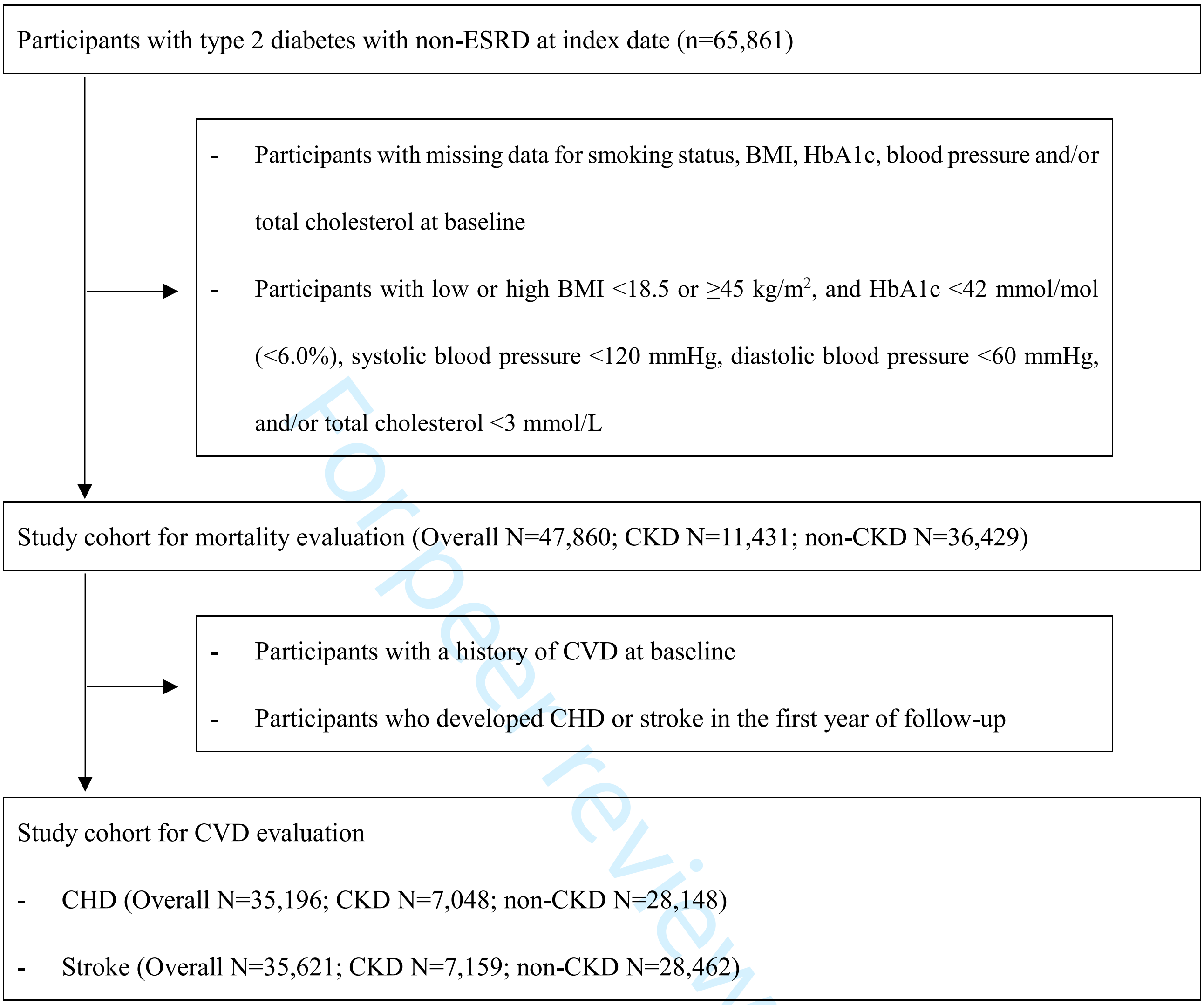

BMI, body mass index; CKD, chronic kidney disease; CHD, coronary heart disease; CVD, Cardiovascular diseases; eGFR, estimated glomerular filtration rate; ESRD, end-stage renal disease; RRT, renal replacement therapy

For peer review only

Table S1. Breakdown of risk factors controlled according to chronic kidney disease and a history of cardiovascular diseases

Number of risk factors controlled	HbA1c <53 mmol/mol (<7.0%)	Blood pressure <140/90 mmHg	Total cholesterol <5 mmol/L	No smoking	CKD			Non-CKD		
					Overall (N=11,431)	No CVD (N=7,216)	CVD (N=4,215)	Overall (N=36,429)	No CVD (N=28,569)	CVD (N=7,860)
0	–	–	–	–	138 (1)	87 (1)	51 (1)	806 (2)	678 (2)	128 (2)
1	Y	–	–	–	69 (0.6)	46 (0.6)	23 (0.6)	312 (1)	260 (0.9)	52 (0.7)
	–	Y	–	–	89 (0.8)	64 (0.9)	25 (0.6)	759 (2)	624 (2)	135 (2)
	–	–	Y	–	438 (4)	264 (4)	174 (4)	1,742 (5)	1,350 (5)	392 (5)
	–	–	–	Y	831 (7)	597 (8)	234 (6)	2,559 (7)	2,187 (8)	372 (5)
2	Y	Y	–	–	62 (0.5)	43 (0.6)	19 (0.5)	304 (0.8)	268 (0.9)	36 (0.5)
	Y	–	Y	–	245 (2)	152 (2)	93 (2)	794 (2)	590 (2)	204 (3)
	Y	–	–	Y	407 (4)	289 (4)	118 (3)	1,070 (3)	911 (3)	159 (2)
	–	Y	Y	–	390 (3)	215 (3)	175 (4)	1,911 (5)	1,446 (5)	465 (6)
	–	Y	–	Y	531 (5)	379 (5)	152 (4)	2,205 (6)	1,910 (7)	295 (4)
	–	–	Y	Y	2,527 (22)	1,615 (22)	912 (22)	7,004 (19)	5,477 (19)	1,527 (19)
3	Y	Y	Y	–	247 (2)	152 (2)	95 (2)	1,073 (3)	788 (3)	285 (4)
	Y	Y	–	Y	302 (3)	199 (3)	103 (2)	1,067 (3)	905 (3)	162 (2)
	Y	–	Y	Y	1,552 (14)	1,019 (14)	533 (13)	3,487 (10)	2,678 (9)	809 (10)
	–	Y	Y	Y	2,139 (19)	1,228 (17)	911 (22)	7,030 (19)	5,294 (19)	1,736 (22)
4	Y	Y	Y	Y	1,464 (13)	867 (12)	597 (14)	4,306 (12)	3,203 (11)	1,103 (14)

Y, meeting the criterion; –, not meeting the criterion

CKD, chronic kidney disease; CVD, (a history of) cardiovascular diseases

For peer review only

For peer review only - http://bmjopen.bmj.com/site/about/guidelines.xhtml

Figure S2. Relative hazards of individual risk factors controlled for mortality and cardiovascular events in (a) participants with chronic kidney disease (CKD) and (b) participants with non-CKD. Hazard ratios (HRs) for all-cause mortality and subdistribution hazard ratios (SHRs) for cardiovascular mortality, coronary heart disease, and stroke were adjusted for age, gender, CKD stage (for CKD cohort), body mass index, deprivation level, duration of diabetes, proteinuria status, a history of cardiovascular diseases (for mortality evaluation), prescribing of antidiabetic, antihypertensive, statins and antiplatelet drugs, and index year.

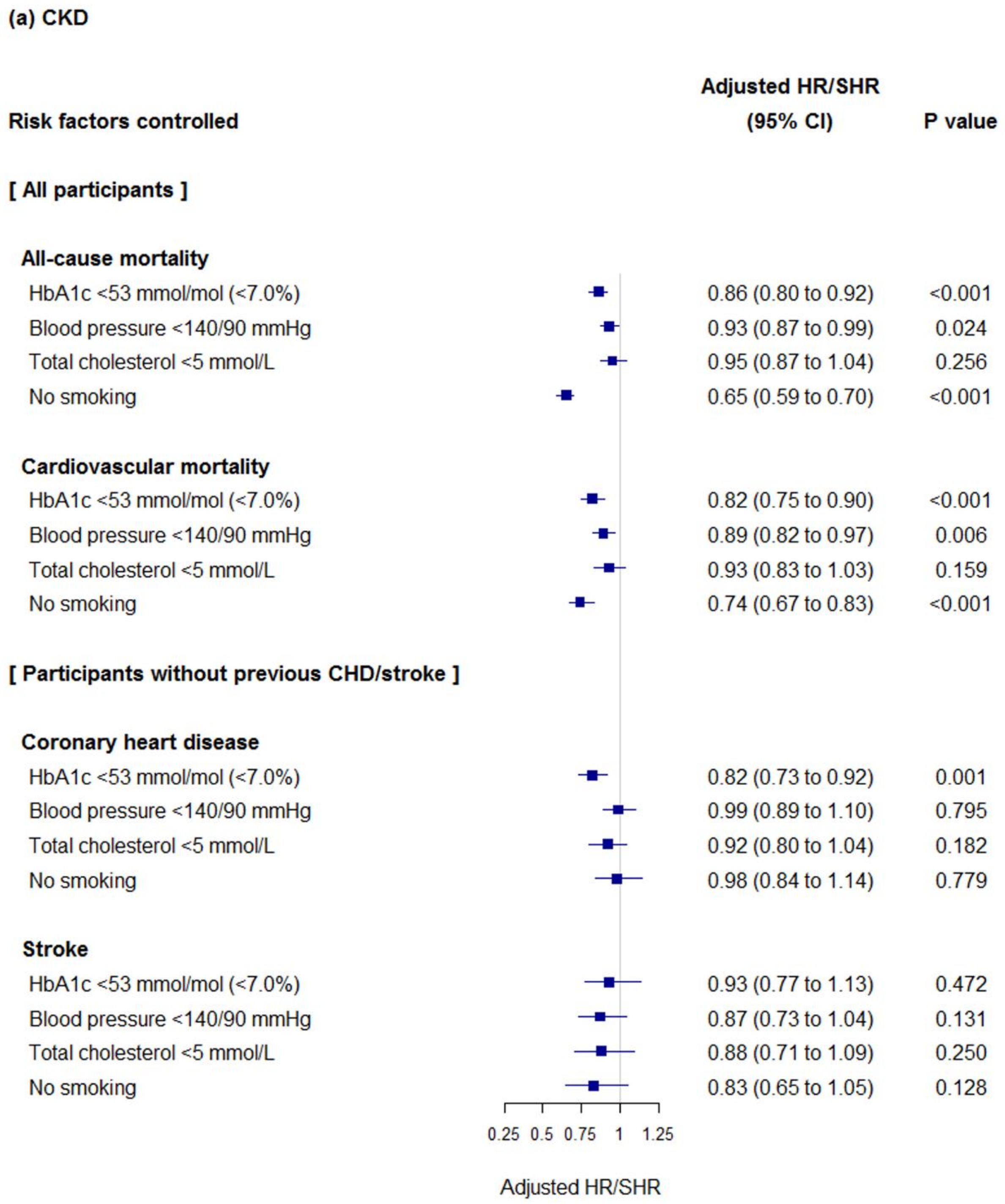

For peer review only - http://bmjopen.bmj.com/site/about/guidelines.xhtml

(b) Non-CKD

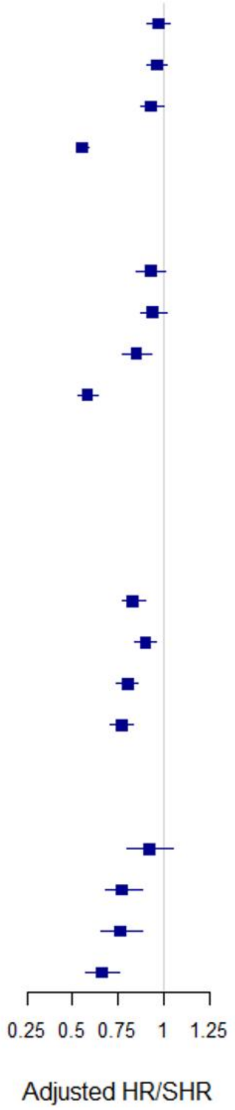

Risk factors controlled	Adjusted HR/SHR (95% CI)	P value
**[All participants]**		
**All-cause mortality**		
HbA1c <53 mmol/mol (<7.0%)	0.97 (0.91 to 1.03)	0.299
Blood pressure <140/90 mmHg	0.96 (0.91 to 1.02)	0.181
Total cholesterol <5 mmol/L	0.93 (0.87 to 1.00)	0.055
No smoking	0.55 (0.52 to 0.59)	<0.001
**Cardiovascular mortality**		
HbA1c <53 mmol/mol (<7.0%)	0.93 (0.85 to 1.01)	0.076
Blood pressure <140/90 mmHg	0.94 (0.87 to 1.02)	0.119
Total cholesterol <5 mmol/L	0.85 (0.77 to 0.93)	0.001
No smoking	0.58 (0.53 to 0.64)	<0.001
**[Participants without previous CHD/stroke]**		
**Coronary heart disease**		
HbA1c <53 mmol/mol (<7.0%)	0.83 (0.77 to 0.90)	<0.001
Blood pressure <140/90 mmHg	0.90 (0.84 to 0.96)	0.001
Total cholesterol <5 mmol/L	0.80 (0.74 to 0.86)	<0.001
No smoking	0.77 (0.71 to 0.83)	<0.001
**Stroke**		
HbA1c <53 mmol/mol (<7.0%)	0.92 (0.80 to 1.05)	0.207
Blood pressure <140/90 mmHg	0.77 (0.68 to 0.88)	<0.001
Total cholesterol <5 mmol/L	0.76 (0.66 to 0.88)	<0.001
No smoking	0.66 (0.57 to 0.76)	<0.001

For peer review only - http://bmjopen.bmj.com/site/about/guidelines.xhtml

STROBE 2007 (v4) Statement—Checklist of items that should be included in reports of *cohort studies*

Section/Topic	Item #	Recommendation	Reported on page #
**Title and abstract**	1	(*a*) Indicate the study's design with a commonly used term in the title or the abstract	1,2
		(*b*) Provide in the abstract an informative and balanced summary of what was done and what was found	2,3
**Introduction**			
Background/rationale	2	Explain the scientific background and rationale for the investigation being reported	5,6
Objectives	3	State specific objectives, including any prespecified hypotheses	6
**Methods**			
Study design	4	Present key elements of study design early in the paper	6,7
Setting	5	Describe the setting, locations, and relevant dates, including periods of recruitment, exposure, follow-up, and data collection	6,7
Participants	6	(*a*) Give the eligibility criteria, and the sources and methods of selection of participants. Describe methods of follow-up	7,8,10
		(*b*) For matched studies, give matching criteria and number of exposed and unexposed	NA
Variables	7	Clearly define all outcomes, exposures, predictors, potential confounders, and effect modifiers. Give diagnostic criteria, if applicable	9
Data sources/ measurement	8*	For each variable of interest, give sources of data and details of methods of assessment (measurement). Describe comparability of assessment methods if there is more than one group	6-9
Bias	9	Describe any efforts to address potential sources of bias	10,11
Study size	10	Explain how the study size was arrived at	6-8
Quantitative variables	11	Explain how quantitative variables were handled in the analyses. If applicable, describe which groupings were chosen and why	11
Statistical methods	12	(*a*) Describe all statistical methods, including those used to control for confounding	11
		(*b*) Describe any methods used to examine subgroups and interactions	11
		(*c*) Explain how missing data were addressed	8,11
		(*d*) If applicable, explain how loss to follow-up was addressed	10
		(*e*) Describe any sensitivity analyses	NA
**Results**			

For peer review only

Participants	13*	(a) Report numbers of individuals at each stage of study—eg numbers potentially eligible, examined for eligibility, confirmed eligible, included in the study, completing follow-up, and analysed	12,Figure S1
		(b) Give reasons for non-participation at each stage	12,Figure S1
		(c) Consider use of a flow diagram	Figure S1
Descriptive data	14*	(a) Give characteristics of study participants (eg demographic, clinical, social) and information on exposures and potential confounders	12,Table 1
		(b) Indicate number of participants with missing data for each variable of interest	Table 1,Figure S1
		(c) Summarise follow-up time (eg, average and total amount)	Figure 1
Outcome data	15*	Report numbers of outcome events or summary measures over time	Figure 1
Main results	16	(*a*) Give unadjusted estimates and, if applicable, confounder-adjusted estimates and their precision (eg, 95% confidence interval). Make clear which confounders were adjusted for and why they were included	12-15
		(*b*) Report category boundaries when continuous variables were categorized	9,11
		(*c*) If relevant, consider translating estimates of relative risk into absolute risk for a meaningful time period	NA
Other analyses	17	Report other analyses done—eg analyses of subgroups and interactions, and sensitivity analyses	NA
**Discussion**			
Key results	18	Summarise key results with reference to study objectives	13-14
**Limitations**			
Interpretation	20	Give a cautious overall interpretation of results considering objectives, limitations, multiplicity of analyses, results from similar studies, and other relevant evidence	15-18
Generalisability	21	Discuss the generalisability (external validity) of the study results	16-18
**Other information**			
Funding	22	Give the source of funding and the role of the funders for the present study and, if applicable, for the original study on which the present article is based	19

*Give information separately for cases and controls in case-control studies and, if applicable, for exposed and unexposed groups in cohort and cross-sectional studies.

Note: An Explanation and Elaboration article discusses each checklist item and gives methodological background and published examples of transparent reporting. The STROBE checklist is best used in conjunction with this article (freely available on the Web sites of PLoS Medicine at http://www.plosmedicine.org/, Annals of Internal Medicine at http://www.annals.org/, and Epidemiology at http://www.epidem.com/). Information on the STROBE Initiative is available at www.strobe-statement.org.

For peer review only

Multiple risk factor control, mortality and cardiovascular events in type 2 diabetes and chronic kidney disease: a population-based cohort study

Journal:	*BMJ Open*
Manuscript ID	bmjopen-2017-019950.R2
Article Type:	Research
Date Submitted by the Author:	26-Mar-2018
Complete List of Authors:	Hamada, Shota; Institute for Health Economics and Policy, Research Department<br>Gulliford, Martin; King's College London, UK,
**Primary Subject Heading**:	Diabetes and endocrinology
Secondary Subject Heading:	General practice / Family practice
Keywords:	cardiovascular diseases, chronic kidney disease, mortality, type 2 diabetes

SCHOLARONE™
Manuscripts

For peer review only

Multiple risk factor control, mortality and cardiovascular events in type 2 diabetes and chronic kidney disease: a population-based cohort study

Shota Hamada[1,2], Martin C Gulliford[1,3]

1 Department of Primary Care and Public Health Sciences, King's College London, London, UK

2 Research Department, Institute for Health Economics and Policy, Association for Health Economics Research and Social Insurance and Welfare, Tokyo, Japan

3 National Institute for Health Research Biomedical Research Centre at Guy's and St Thomas' National Health Service Foundation Trust, London UK

Correspondence to: Shota Hamada

Research Department, Institute for Health Economics and Policy, Association for Health Economics Research and Social Insurance and Welfare

No.11 Toyo-Kaiji Bldg, 1-5-11 Nishi-Shimbashi, Minato-ku, Tokyo, 105-0003 Japan

Tel: +81 3-3506-8529, Fax: +81 3-3506-8528, E-mail: shota.hamada@ihep.jp

For peer review only

Abstract

OBJECTIVES: This study aimed to evaluate the effectiveness of multiple risk factor control (MRFC) at reducing mortality and cardiovascular events in diabetes and chronic kidney disease (CKD) in clinical practice.

DESIGN: Population-based cohort study.

SETTING: Primary care database in the UK, linked with inpatient and mortality data.

PARTICIPANTS: Participants aged 40 to 79 years with type 2 diabetes and valid serum creatinine measurements, including 11,431 participants with CKD (eGFR 15–59 mL/min/1.73 m^2) and 36,429 participants with non-CKD (eGFR ≥60 mL/min/1.73 m^2).

EXPOSURES: MRFC consisted of four components: HbA1c <53 mmol/mol (<7.0%), blood pressure <140/90 mmHg, total cholesterol <5 mmol/L, and no smoking. The main exposure variable was the number of risk factors controlled at baseline.

OUTCOME MEASURES: All-cause and cardiovascular mortality in the overall participants. Cardiovascular events, including coronary heart disease and stroke, in participants limited to those without a history of cardiovascular diseases at baseline.

RESULTS: In participants with CKD, 37% or 13% met three or four MRFC criteria, respectively. Increasing numbers of risk factors controlled were associated with lower relative hazards for all outcomes studied compared with those meeting no or one

For peer review only - http://bmjopen.bmj.com/site/about/guidelines.xhtml

criterion. For participants with CKD meeting four criteria, the adjusted hazard ratio (HR) for all-cause mortality was 0.60 (95% confidence interval (CI) 0.53 to 0.69) and the adjusted subdistribution HR for cardiovascular mortality was 0.60 (0.50 to 0.70), considering a competing risk of non-cardiovascular death. Participants meeting four criteria also had lower relative hazards for coronary heart disease (adjusted subdistribution HR 0.73, 95% CI 0.59 to 0.91) and stroke (0.63, 0.45 to 0.89), considering death as a competing risk.

CONCLUSIONS: MRFC may lower the increased risks for mortality and cardiovascular events in people with diabetes and CKD. Further research is needed to evaluate appropriateness of MRFC according to individual participants' health status for improved management of cardiovascular risks in this population.

Strengths and limitations of this study

- This study included a large number of participants with type 2 diabetes and CKD sampled from a representative general population with about 6 years of follow-up, which enabled to determine the associations of cardiovascular risk factors with mortality and cardiovascular events.
- Linked data for diagnostic data in hospitals and death registration with a primary

For peer review only

care database enhanced the validity of the study to evaluate mortality and cardiovascular events.

- We could not conclude that associations represented causal relationships between MRFC and mortality and cardiovascular events in this non-randomised study.
- There is a possibility of confounding if healthier participants were managed more successfully and this resulted in being categorised as those with greater number of risk factors controlled.

For peer review only

For peer review only - http://bmjopen.bmj.com/site/about/guidelines.xhtml

Introduction

Diabetes and chronic kidney disease (CKD) are growing health problems worldwide, contributing to increased mortality [1]. Diabetes and CKD also impose a substantial economic burden on society, with particularly high costs relating to cardiovascular complications and renal replacement therapy [2,3]. The prevalence of CKD in patients with diabetes is between 4.2% and 17.9% (CKD stages 3 to 5) in European countries [4]. The leading cause of death in people with type 2 diabetes or CKD is cardiovascular disease rather than renal complications [5,6]. Prevention of cardiovascular events is a key focus in the management of patients with these conditions.

Multifactorial interventions to reduce cardiovascular risks were shown to be effective at reducing mortality and cardiovascular events in patients with type 2 diabetes and persistent microalbuminuria in the Steno-2 randomised trial [7,8]. This study provided a high level of evidence, but included a relatively small number of participants with diabetes who were managed in specialist centres. Recently, the implementation and effectiveness of this approach have been evaluated in patients with diabetes in clinical practice settings [9-11]. Epidemiological studies have demonstrated additional risks of CKD on mortality and cardiovascular diseases in people with diabetes [12], but

For peer review only - http://bmjopen.bmj.com/site/about/guidelines.xhtml

treatment approaches in this population have not been well studied. No studies focused on multiple risk factor control (MRFC) in patients with both diabetes and CKD in a wide clinical practice setting. Generally, patients with kidney disease have been underrepresented in cardiovascular clinical trials [13]. This population may have an altered risk-benefit profile, and extrapolation of data based on patients with normal kidney function into patients with CKD may be unreliable [13]. We aimed to conduct a pragmatic evaluation of the effectiveness of MRFC on mortality and cardiovascular events in participants with type 2 diabetes and CKD in a population-based cohort study.

Methods

Data sources

This study employed a linked dataset derived from the UK Clinical Practice Research Datalink (CPRD), the UK National Health Service Hospital Episodes Statistics (HES) inpatient data, and the UK Office for National Statistics (ONS) mortality data. The CPRD contains anonymised electronic health records from general practices across the UK [14]. The CPRD collects data for diagnoses and clinical assessment, prescriptions and laboratory test results, such as HbA1c and serum creatinine. The HES inpatient data were comprised of inpatient records from all National Health Service hospitals in

For peer review only
For peer review only - http://bmjopen.bmj.com/site/about/guidelines.xhtml

England. Information on the date of death and the causes of death were available in the ONS mortality data file. Multiple causes of death can be recorded in the mortality data. Diagnoses and clinical evaluation in the CPRD were coded with the Read codes, a hierarchical coding system used in primary care in the UK, whereas those in the HES and ONS were coded with the International Classification of Diseases, tenth revision (ICD-10). Linked data are available for general practices in England only and participants were limited to those with linked data for the HES and ONS available. The study was approved by the CPRD Independent Scientific Advisory Committee (ISAC Protocol 15_201R).

Study population

The scheme of the study cohort selection is presented in figure S1. We initially sampled participants who were diagnosed with type 2 diabetes from the CPRD [15]. Using the CPRD records, the date of the first valid serum creatinine value between 2006 and 2010 recorded more than one year after the first diagnosis of diabetes were defined as the index date. A similar approach was taken by Adamsson Eryd et al [16] to ensure that participants managed for diabetes had sufficient time available for recording of baseline values. To avoid misclassification of CKD status and stage, the index serum creatinine

For peer review only - http://bmjopen.bmj.com/site/about/guidelines.xhtml

values were validated by confirmation of subsequent values within 30% of the index values. We restricted the sample to participants aged 40 to 79 years at the index date with at least one year of follow-up data available (ie, participants who died in the first year of follow-up were excluded). Estimated glomerular filtration rate (eGFR) was calculated from a serum creatinine value, age, gender, and ethnicity, using the Chronic Kidney Disease Epidemiology Collaboration (CKD-EPI) equation [17]. Missing ethnicity was assumed as 'non-black' in the present study. Participants diagnosed with end-stage renal disease, those who had received renal replacement therapy, or those with index eGFR <15 mL/min/1.73 m^2 were excluded. We also excluded participants with missing data for smoking status, body mass index (BMI), HbA1c, blood pressure, or total cholesterol, or those with extreme BMI (<18.5 or ≥45 kg/m^2) at baseline. Since it has been reported that low values of cardiovascular risk factors were not always associated with better outcomes in observational studies [15,18,19], possibly due to reverse causation [20,21], participants with low HbA1c (<42 mmol/mol or <6.0%), blood pressure (systolic <120 or diastolic <60 mmHg), and total cholesterol (<3 mmol/L) were further excluded. Participants were categorised according to index eGFR into participants with CKD (<60 mL/min/1.73 m^2) and those with non-CKD (≥60 mL/min/1.73 m^2).

For peer review only - http://bmjopen.bmj.com/site/about/guidelines.xhtml

Multiple risk factor control

MRFC was defined in this study as consisting of four components: (1) HbA1c <53 mmol/mol (<7.0%), (2) blood pressure <140/90 mmHg (systolic <140 and diastolic <90 mmHg), (3) total cholesterol <5 mmol/L, and (4) no smoking (non- or ex-smokers). The means of HbA1c, blood pressure, and total cholesterol records within one year before the index date were evaluated. The number of the risk factors controlled from four criteria was treated as the exposure and included as a categorical variable in the analyses, with those meeting no or one criterion as a reference category.

Outcomes

Main outcomes of interest in this study included all-cause and cardiovascular mortality, fatal and non-fatal coronary heart disease (CHD) and stroke. The date of death and causes of death were determined using the ONS mortality data. Participants who died from cardiovascular causes were identified if people had any of the ICD-10 codes I00 to I99 as a cause of death. Similarly, participants who died from renal causes were identified by the ICD-10 codes N17 to N19. All of the CPRD, HES and ONS were used to ascertain fatal and non-fatal CHD and stroke. Read codes for CHD and stroke

For peer review only - http://bmjopen.bmj.com/site/about/guidelines.xhtml

reported previously [22,23] were updated for the present study. The ICD-10 codes for CHD and stroke were I20 to I25 and I60, I61, I63 and I64, respectively.

Analysis

Baseline characteristics of the study cohort were described according to CKD status. Time-to-event analyses were conducted to evaluate the associations of MRFC with mortality and cardiovascular events. To address the issue of reverse causation and to avoid misclassification of the outcomes from those which had existed at baseline, person-years for participants who experienced outcomes of interest in the first year of follow-up were excluded from analyses (figure S1). Cox proportional hazards models were used to evaluate the association of MRFC with all-cause mortality. Proportional hazards assumption was assessed by visual inspection of log-log plots, and no apparent violation was found. Competing risks regression with subdistribution hazard models were conducted for cardiovascular mortality and cardiovascular events, considering competing risks for non-cardiovascular and all-cause death, respectively [24]. Associations of MRFC with cardiovascular events were evaluated in participants without a known history of cardiovascular diseases at baseline (figure S1). Participants were followed from the index date until the earliest of the events of interest, the last date

For peer review only - http://bmjopen.bmj.com/site/about/guidelines.xhtml

of CPRD records, or 31 March 2015 for all-cause mortality evaluation. In the competing risks regression analyses for cardiovascular mortality and cardiovascular events, participants who experienced the corresponding competing events prior to the event of interest were also censored.

Main analyses were conducted by CKD status, adjusting for a range of baseline covariates, including age (continuous), gender (male or female), CKD stage (3a, 3b, and 4; for CKD cohort), BMI (18.5–24.9, 25.0–29.9, 30.0–34.9, 35.0–39.9, and 40.0–44.9 kg/m^2), deprivation level (quintile; 1, least deprived, to 5, most deprived), duration of diabetes (1.0–4.9, 5.0–9.9, and 10+ years), proteinuria status, including microalbuminuria (yes, no, and a missing category), a history of cardiovascular diseases, including CHD and stroke (for mortality evaluation), and prescribing during six months prior to the index date of antidiabetic drugs (none, insulin with and without other antidiabetic drugs, and non-insulin drugs only), antihypertensive drugs (none, drugs acting on renin-angiotensin system with and without other antihypertensive drugs, and other classes of antihypertensive drugs only, including β-blockers, calcium channel blockers, and thiazide diuretics), statins and antiplatelet drugs, and index year (2006 to 2010). In addition, the associations of CKD with the outcomes were evaluated

For peer review only - http://bmjopen.bmj.com/site/about/guidelines.xhtml

according to the number of risk factors controlled, adjusting for the potential confounding factors described above.

In this paper, the results for participants with CKD were focused on, with the results for those with non-CKD shown for comparative purposes. The associations of each component of MRFC with the outcomes were also evaluated to aid interpretation of the study results. All analyses were performed using Stata version 14 (Stata Corp., College Station TX). The 'forestplot' package in R was used to present the results [25].

Patient and Public Involvement

No patients were involved in setting the research question or the outcome measures, nor were they involved in developing plans for design or implementation of the study. No patients were asked to advise on interpretation or writing up of results. Results will be disseminated to relevant patient communities through news media.

Results

Characteristics of the study population

Baseline characteristics of the study cohort are shown according to CKD status in table

For peer review only - http://bmjopen.bmj.com/site/about/guidelines.xhtml

1. Mean index eGFR was 49 mL/min/1.73 m^2 for participants with CKD and 81 mL/min/1.73 m^2 for those with non-CKD. Participants with CKD were older (71 *vs* 62 years), included more women (52% *vs* 40%), had a longer duration of diabetes, and were more likely to have a history of cardiovascular diseases (37% *vs* 22%). A higher frequency of proteinuria was recorded in participants with CKD (18% *vs* 12% among participants with records of proteinuria status). HbA1c and total cholesterol were slightly lower in participants with CKD. Although diastolic blood pressure was lower in participants with CKD, systolic blood pressure was higher despite more people under antihypertensive medications. Participants with CKD were prescribed insulin, drugs on renin-angiotensin system, statins, and antiplatelet drugs more frequently.

Implementation of MRFC

The number of risk factors controlled from four components of MRFC are shown in table 2. More detailed results of which of the components were controlled are available in table S1. Higher rates of control for HbA1c, total cholesterol, and smoking status were observed in participants with CKD compared with those with non-CKD. However, blood pressure was less likely managed in participants with CKD (46% *vs* 51%). There were some differences in management status according to a history of cardiovascular

For peer review only - http://bmjopen.bmj.com/site/about/guidelines.xhtml

diseases; in participants with CKD, higher rates of control of blood pressure (49% *vs* 44%) and total cholesterol (83% *vs* 76%) in participants with a history of cardiovascular diseases compared with those without. Participants meeting three or four criteria accounted for 37% or 13% in participants with CKD.

Effectiveness of MRFC

Absolute risks for mortality and cardiovascular diseases and adjusted relative hazards of the number of risk factors controlled for the outcomes are shown in figure 1. Increasing numbers of risk factors controlled were associated with lower relative hazards for all outcomes studied relative to participants meeting no or one criterion. For participants with CKD meeting four MRFC criteria, the adjusted hazard ratio (HR) for all-cause mortality was 0.60 (95% CI 0.53 to 0.69), and adjusted subdistribution HR for cardiovascular mortality was 0.60 (0.50 to 0.70). Participants meeting four criteria also had lower relative risks for CHD (adjusted subdistribution HR 0.73, 95% CI 0.59 to 0.91) and stroke (0.63, 0.45 to 0.89) in participants with CKD. In participants with non-CKD, increasing numbers of risk factors controlled were also associated with lower risks for all-cause and cardiovascular mortality, CHD, and stroke. As shown in figure S2, the strengths of associations of each component of MRFC with mortality and

For peer review only - http://bmjopen.bmj.com/site/about/guidelines.xhtml

cardiovascular diseases were different; for example, the greatest associations of no smoking with all-cause and cardiovascular mortality were observed in participants with and without CKD.

Comparisons between CKD and non-CKD

Unadjusted absolute risks for mortality and cardiovascular diseases were higher in participants with CKD by 1.4- to 2.9-fold compared with those with non-CKD at the same MRFC category (figure 1). More participants with CKD died from cardiovascular causes compared with those without (63% *vs* 54%, P<0.001). More participants with CKD died from renal causes (n=631 or 5% *vs* n=326 or 0.9%, P<0.001), but the proportions were much smaller than cardiovascular causes of death. Relative hazards of CKD for the outcomes are shown in figure 2. After adjustment with possible confounding factors, comorbid CKD remained to be associated with greater risks for all-cause mortality (adjusted HR, 1.16 to 1.30) and cardiovascular mortality (adjusted subdistribution HR, 1.25 to 1.41). In participants meeting two or more criteria, comorbid CKD was associated with a higher risk for CHD (1.18 to 1.25). The associations of comorbid CKD with stroke was observed in participants meeting four criteria only (1.64).

For peer review only - http://bmjopen.bmj.com/site/about/guidelines.xhtml

Discussion

In this population-based cohort study of participants with type 2 diabetes and CKD stages 3 or 4, MRFC was associated with lower relative risks for mortality (N>11,000) and cardiovascular diseases (N>7,000). We also confirmed that CKD was associated with increased risks for mortality and cardiovascular events. Higher absolute risks for mortality and cardiovascular events and great relative risk reduction associated with MRFC suggest that the MRFC strategy may be one of the main approaches to potentially reducing the burden of diabetes and CKD.

This study evaluated the effectiveness of MRFC in patients with type 2 diabetes according to presence or absence of CKD in clinical practice. So far, the associations of MRFC with lower risks for mortality and cardiovascular events have been shown in people with diabetes, not focusing on CKD status. Participants with controlled three risk factors of HbA1c, blood pressure, and LDL cholesterol had 62% and 60% risk reduction for cardiovascular events and CHD, respectively, in patients with diabetes without known cardiovascular diseases [10]. The associations of uncontrolled HbA1c, blood pressure, LDL cholesterol, and smoking with mortality and cardiovascular events were

For peer review only - http://bmjopen.bmj.com/site/about/guidelines.xhtml

individually evaluated in a large population-based study with >850,000 participants with diabetes [11]. The study cohort included 35.5% of participants with CKD in those with cardiovascular diseases and 21.8% in those without, and CKD was included in the analyses for adjustment. This study suggested that uncontrolled risk factors attributed to about 1 in 3 major cardiovascular events and fewer 1 in 10 deaths.

The strength of this study was the inclusion of a large size of >11,000 participants with diabetes and CKD with an observation of >62,000 person-years. In addition to the large sample size and long-term follow-up, representativeness from general population and data quality are also advantages of the CPRD [14], which should remain even if linked data for HES and ONS are only available for England practices. Instead, linked data for diagnoses in hospitals and death registration substantially enhanced the validity of the study to evaluate mortality and cardiovascular events.

There are also some limitations in this study. First, despite our focus on the number of MRFC, the impacts of each component of MRFC on mortality and cardiovascular events were different. Different cut-off points for HbA1c, blood pressure, and total cholesterol may bring different results. Next, we could not conclude that associations

For peer review only - http://bmjopen.bmj.com/site/about/guidelines.xhtml

represented causal relationships between MRFC and mortality and cardiovascular events in this non-randomised study. There is a possibility of confounding if healthier participants were managed more successfully and this resulted in being categorised as those with greater number of risk factors controlled. For example, stringent management of HbA1c might not be targeted for vulnerable participants due to concerns for greater risk of hypoglycaemia, a form of confounding by contra-indication. We cannot exclude the possibility of residual confounding, despite adjustment with a range of covariates in the analyses, including physical activity and alcohol intake [26,27]. Then, measurement and assay methods for HbA1c, blood pressure, cholesterol and serum creatinine might not have been standardised among general practices or laboratories. As well as missing data on ethnicity and fluctuations in serum creatinine values, these methodological limitations might influence the determination of CKD status or staging. Although proteinuria has been known as a risk factor for mortality and cardiovascular diseases [28,29], we could not determine proteinuria status completely as reported previously [30,31]. Incomplete records on proteinuria may introduce a bias for proteinuria status and possibly influence the study results. Finally, although we used one of the largest primary care electronic health records database, it seemed to be insufficient to separately evaluate MRFC for participants with different stages of CKD.

For peer review only - http://bmjopen.bmj.com/site/about/guidelines.xhtml

Further research is needed to focus on patients with more advanced CKD who may have altered risk-benefit profile compared with patients with less impaired renal function.

In summary, based on the population-based cohort study of routine clinical practices, MRFC may lower the increased risks for mortality and cardiovascular events in people with diabetes and CKD. Further research is needed to evaluate appropriateness of MRFC according to individual participants' health status for improved management of cardiovascular risks in this population.

Acknowledgments

SH was supported by the Uehara Memorial Foundation Postdoctoral Fellowship, Tokyo, Japan. MCG was supported by the National Institute for Health Research (NIHR) Biomedical Research Centre at Guy's and St Thomas' NHS Foundation Trust and King's College London. This study is based on data from the CPRD obtained under license from the UK Medicines and Healthcare products Regulatory Agency. However, the interpretation and conclusions contained in this report are those of the authors alone and not necessarily those of the National Health Service, the NIHR or the Department of Health.

For peer review only - http://bmjopen.bmj.com/site/about/guidelines.xhtml

Contributors: Both authors contributed to conception and study design of the study, data acquisition, statistical analysis, and interpretation. SH drafted the manuscript and MCG revised it critically for important intellectual content.

Funding: This research received no specific grant from any funding agency.

Competing interests: None declared.

Provenance and peer review: Not commissioned; externally peer reviewed.

Data sharing statement: No additional data are available.

For peer review only

References

1. GBD 2013 Mortality and Causes of Death Collaborators. Global, regional, and national age-sex specific all-cause and cause-specific mortality for 240 causes of death, 1990-2013: a systematic analysis for the Global Burden of Disease Study 2013. *Lancet.* 2015;385(9963):117-171.

2. Seuring T, Archangelidi O, Suhrcke M. The Economic Costs of Type 2 Diabetes: A Global Systematic Review. *Pharmacoeconomics.* 2015;33(8):811-831.

3. Kerr M, Bray B, Medcalf J, O'Donoghue DJ, Matthews B. Estimating the financial cost of chronic kidney disease to the NHS in England. *Nephrol Dial*

For peer review only - http://bmjopen.bmj.com/site/about/guidelines.xhtml

Transplant. 2012;27 Suppl 3:iii73-80.

4. Brück K, Stel VS, Gambaro G, Hallan S, Völzke H, Ärnlöv J, et al. CKD Prevalence Varies across the European General Population. *J Am Soc Nephrol.* 2016;27(7):2135-2147.

5. Holman RR, Paul SK, Bethel MA, Matthews DR, Neil HA. 10-year follow-up of intensive glucose control in type 2 diabetes. *N Engl J Med.* 2008;359(15):1577-1589.

6. Go AS, Chertow GM, Fan D, McCulloch CE, Hsu CY. Chronic kidney disease and the risks of death, cardiovascular events, and hospitalization. *N Engl J Med.* 2004;351(13):1296-1305.

7. Gaede P, Vedel P, Larsen N, Jensen GV, Parving HH, Pedersen O. Multifactorial intervention and cardiovascular disease in patients with type 2 diabetes. *N Engl J Med.* 2003;348(5):383-393.

8. Gaede P, Lund-Andersen H, Parving HH, Pedersen O. Effect of a multifactorial intervention on mortality in type 2 diabetes. *N Engl J Med.* 2008;358(6):580-591.

9. Wong ND, Patao C, Wong K, Malik S, Franklin SS, Iloeje U. Trends in control of cardiovascular risk factors among US adults with type 2 diabetes from 1999 to 2010: comparison by prevalent cardiovascular disease status. *Diab Vasc Dis Res.*

For peer review only - http://bmjopen.bmj.com/site/about/guidelines.xhtml

2013;10(6):505-513.

10. Wong ND, Zhao Y, Patel R, Patao C, Malik S, Bertoni AG, et al. Cardiovascular Risk Factor Targets and Cardiovascular Disease Event Risk in Diabetes: A Pooling Project of the Atherosclerosis Risk in Communities Study, Multi-Ethnic Study of Atherosclerosis, and Jackson Heart Study. *Diabetes Care.* 2016;39(5):668-676.

11. Vazquez-Benitez G, Desai JR, Xu S, Goodrich GK, Schroeder EB, Nichols GA, et al. Preventable major cardiovascular events associated with uncontrolled glucose, blood pressure, and lipids and active smoking in adults with diabetes with and without cardiovascular disease: a contemporary analysis. *Diabetes Care.* 2015;38(5):905-912.

12. Cea Soriano L, Johansson S, Stefansson B, Rodríguez LA. Cardiovascular events and all-cause mortality in a cohort of 57,946 patients with type 2 diabetes: associations with renal function and cardiovascular risk factors. *Cardiovasc Diabetol.* 2015;14:38.

13. Konstantinidis I, Nadkarni GN, Yacoub R, Saha A, Simoes P, Parikh CR, et al. Representation of Patients With Kidney Disease in Trials of Cardiovascular Interventions: An Updated Systematic Review. *JAMA Intern Med.*

For peer review only

For peer review only - http://bmjopen.bmj.com/site/about/guidelines.xhtml

2016;176(1):121-124.

14. Herrett E, Gallagher AM, Bhaskaran K, Forbes H, Mathur R, van Staa T, et al. Data Resource Profile: Clinical Practice Research Datalink (CPRD). *Int J Epidemiol.* 2015;44(3):827-836.

15. Nicholas J, Charlton J, Dregan A, Gulliford MC. Recent HbA1c values and mortality risk in type 2 diabetes. population-based case-control study. *PLoS One.* 2013;8(7):e68008.

16. Adamsson Eryd S, Gudbjörnsdottir S, Manhem K, Rosengren A, Svensson AM, Miftaraj M, et al. Blood pressure and complications in individuals with type 2 diabetes and no previous cardiovascular disease: national population based cohort study. *BMJ.* 2016;354:i4070.

17. Levey AS, Stevens LA, Schmid CH, Zhang YL, Castro AF 3rd, Feldman HI, et al. A new equation to estimate glomerular filtration rate. *Ann Intern Med.* 2009;150(9):604-612.

18. Kontopantelis E, Springate DA, Reeves D, Ashcroft DM, Rutter MK, Buchan I, et al. Glucose, blood pressure and cholesterol levels and their relationships to clinical outcomes in type 2 diabetes: a retrospective cohort study. *Diabetologia.* 2015;58(3):505-518.

For peer review only

For peer review only - http://bmjopen.bmj.com/site/about/guidelines.xhtml

19. Afghahi H, Svensson MK, Pirouzifard M, Eliasson B, Svensson AM. Blood pressure level and risk of major cardiovascular events and all-cause of mortality in patients with type 2 diabetes and renal impairment: an observational study from the Swedish National Diabetes Register. *Diabetologia.* 2015;58(6):1203-1211.

20. Herrington W, Staplin N, Judge PK, Mafham M, Emberson J, Haynes R, et al. Evidence for Reverse Causality in the Association Between Blood Pressure and Cardiovascular Risk in Patients With Chronic Kidney Disease. *Hypertension.* 2017;69(2):314-322.

21. Weiner DE, Sarnak MJ. Managing dyslipidemia in chronic kidney disease. *J Gen Intern Med.* 2004;19(10):1045-1052.

22. Gulliford MC, Charlton J, Ashworth M, Rudd AG, Toschke AM; eCRT Research Team. Selection of medical diagnostic codes for analysis of electronic patient records. Application to stroke in a primary care database. *PLoS One.* 2009;4(9):e7168.

23. Bhattarai N, Charlton J, Rudisill C, Gulliford MC. Coding, recording and incidence of different forms of coronary heart disease in primary care. *PLoS One.* 2012;7(1):e29776.

24. Austin PC, Lee DS, Fine JP. Introduction to the Analysis of Survival Data in the

For peer review only - http://bmjopen.bmj.com/site/about/guidelines.xhtml

Presence of Competing Risks. *Circulation.* 2016;133(6):601-609.

25. Gordon M, Lumley T. Advanced Forest Plot Using 'grid' Graphics. Vienna: The Comprehensive R Archive Network, 2016.

26. Anand SS, Islam S, Rosengren A, Franzosi MG, Steyn K, Yusufali AH, et al. Risk factors for myocardial infarction in women and men: insights from the INTERHEART study. *Eur Heart J.* 2008;29(7):932-940.

27. O'Donnell MJ, Xavier D, Liu L, Zhang H, Chin SL, Rao-Melacini P, et al. Risk factors for ischaemic and intracerebral haemorrhagic stroke in 22 countries (the INTERSTROKE study): a case-control study. *Lancet.* 2010;376(9735):112-123.

28. Chronic Kidney Disease Prognosis Consortium., Matsushita K, van der Velde M, Astor BC, Woodward M, Levey AS, de Jong PE, et al. Association of estimated glomerular filtration rate and albuminuria with all-cause and cardiovascular mortality in general population cohorts: a collaborative meta-analysis. *Lancet.* 2010;375(9731):2073-2081.

29. Matsushita K, Coresh J, Sang Y, Chalmers J, Fox C, Guallar E, et al. Estimated glomerular filtration rate and albuminuria for prediction of cardiovascular outcomes: a collaborative meta-analysis of individual participant data. *Lancet Diabetes Endocrinol.* 2015;3(7):514-525.

For peer review only

For peer review only - http://bmjopen.bmj.com/site/about/guidelines.xhtml

30. Dreyer G, Hull S, Aitken Z, Chesser A, Yaqoob MM. The effect of ethnicity on the prevalence of diabetes and associated chronic kidney disease. *QJM.* 2009;102(4):261-269.

31. Liang H, Kennedy C, Manne S, Lin JH, Dolin P. Monitoring for proteinuria in patients with type 2 diabetes mellitus. *BMJ Open Diabetes Res Care.* 2015;3(1):e000071.

For peer review only

Table 1. Baseline characteristics of the study cohort by CKD status

		CKD (N=11,431)	Non-CKD (N=36,429)	P value
Age (years)	Mean (SD)	71 (6)	62 (9)	<0.001
Gender	Male	5,481 (48)	22,006 (60)	<0.001
	Female	5,950 (52)	14,423 (40)	
eGFR (mL/min/1.73 m^2)	Mean (SD)	49 (9)	81 (13)	–
	15–29	558 (5)	–	
	30–44	2,655 (23)	–	
	45–59	8,218 (72)	–	
Smoking status	Non-smoker	5,426 (47)	16,511 (45)	<0.001
	Ex-smoker	4,327 (38)	12,217 (34)	
	Current smoker	1,678 (15)	7,701 (21)	
BMI (kg/m^2)	18.5–24.9	1,459 (13)	4,097 (11)	<0.001
	25.0–29.9	4,329 (38)	13,054 (36)	
	30.0–34.9	3,527 (31)	11,485 (32)	
	35.0–39.9	1,541 (13)	5,454 (15)	
	40.0–44.9	575 (5)	2,339 (6)	
Deprivation level (quintile)	1 (least deprived)	1,508 (13)	4,785 (13)	0.293
	2	2,331 (20)	7,300 (20)	
	3	2,374 (21)	7,640 (21)	
	4	2,637 (23)	8,172 (22)	
	5 (most deprived)	2,581 (23)	8,532 (23)	
Duration of diabetes (years)	1.0–4.9	5,208 (46)	22,527 (62)	<0.001
	5.0–9.9	2,954 (26)	8,356 (23)	
	≥10.0	3,269 (29)	5,546 (15)	
Proteinuria	Yes	1,714 (15)	3,279 (9)	<0.001
	No	7,666 (67)	24,110 (66)	
	Missing	2,051 (18)	9,040 (25)	
History of coronary heart disease and/or stroke		4,215 (37)	7,860 (22)	<0.001
HbA1c (mmol/mol or %)	42–47 (6.0–6.4)*	1,307 (11)	3,513 (10)	<0.001
	48–52 (6.5–6.9)	3,041 (27)	8,900 (24)	
	53–57 (7.0–7.4)	2,590 (23)	7,781 (21)	
	58–63 (7.5–7.9)	1,709 (15)	5,461 (15)	

For peer review only - http://bmjopen.bmj.com/site/about/guidelines.xhtml

	64–68 (8.0–8.4)	1,038 (9)	3,567 (10)	
	≥69 (≥8.5)	1,746 (15)	7,207 (20)	
Systolic blood pressure (mmHg)	120–129	1,777 (16)	7,203 (20)	<0.001
	130–139	3,508 (31)	12,121 (33)	
	140–149	3,387 (30)	10,242 (28)	
	≥150	2,759 (24)	6,863 (19)	
Diastolic blood pressure (mmHg)	60–79	7,238 (63)	16,803 (46)	<0.001
	80–89	3,599 (31)	15,816 (43)	
	≥90	594 (5)	3,810 (10)	
Total cholesterol (mmol/L)	3.0–3.9	3,782 (33)	10,960 (30)	<0.001
	4.0–4.9	5,220 (46)	16,387 (45)	
	≥5.0	2,429 (21)	9,082 (25)	
Medication	Antidiabetic drugs			<0.001
	Insulin (± non-insulin)	1,805 (16)	3,225 (9)	
	Non-insulin only	7,722 (68)	26,753 (73)	
	Antihypertensive drugs			<0.001
	Drugs on renin-angiotensin system (± others)	8,472 (74)	21,535 (59)	
	Other antihypertensive drugs only	1,610 (14)	4,751 (13)	
	Statins	9,004 (79)	27,011 (74)	<0.001
	Antiplatelet drugs	6,440 (56)	16,375 (45)	<0.001
Index year	2006	9,091 (80)	24,192 (66)	<0.001
	2007	1,008 (9)	3,741 (10)	
	2008	545 (5)	2,880 (8)	
	2009	432 (4)	2,677 (7)	
	2010	355 (3)	2,939 (8)	

BMI, body mass index; CKD, chronic kidney disease; eGFR, estimated glomerular filtration rate

Frequencies (percentages) are shown otherwise specified.

* Participants with HbA1c <48 mmol/mol (<6.5%) were only included if they were prescribed antidiabetic drugs.

For peer review only - http://bmjopen.bmj.com/site/about/guidelines.xhtml

Table 2. Risk factors controlled according to chronic kidney disease and a history of cardiovascular diseases

	CKD			Non-CKD		
	Total (N=11,431)	No CVD (N=7,216)	CVD (N=4,215)	Total (N=36,429)	No CVD (N=28,569)	CVD (N=7,860)
Individual risk factor controlled						
HbA1c <53 mmol/mol (<7.0%)	4,348 (38)	2,767 (38)	1,581 (38)	12,413 (34)	9,603 (34)	2,810 (36)
Blood pressure <140 & <90 mmHg	5,224 (46)	3,147 (44)	2,077 (49)	18,655 (51)	14,438 (51)	4,217 (54)
Total cholesterol <5 mmol/L	9,002 (79)	5,512 (76)	3,490 (83)	27,347 (75)	20,826 (73)	6,521 (83)
No smoking	9,753 (85)	6,193 (86)	3,560 (84)	28,728 (79)	22,565 (79)	6,163 (78)
Number of risk factors controlled						
0	138 (1)	87 (1)	51 (1)	806 (2)	678 (2)	128 (2)
1	1,427 (12)	971 (13)	456 (11)	5,372 (15)	4,421 (15)	951 (12)
2	4,162 (36)	2,693 (37)	1,469 (35)	13,288 (36)	10,602 (37)	2,686 (34)
3	4,240 (37)	2,598 (36)	1,642 (39)	12,657 (35)	9,665 (34)	2,992 (38)
4	1,464 (13)	867 (12)	597 (14)	4,306 (12)	3,203 (11)	1,103 (14)

CKD, chronic kidney disease; CVD, (a history of) cardiovascular diseases

Frequencies (percentages) are shown.

For peer review only

For peer review only - http://bmjopen.bmj.com/site/about/guidelines.xhtml

Figure legends

Figure 1. Relative hazards of the number of risk factors controlled for mortality and cardiovascular events in (a) participants with chronic kidney disease (CKD) and (b) participants with non-CKD. Hazard ratios (HRs) for all-cause mortality and subdistribution hazard ratios (SHRs) for cardiovascular mortality, coronary heart disease, and stroke were adjusted for age, gender, CKD stage (for CKD cohort), body mass index, deprivation level, duration of diabetes, proteinuria status, a history of cardiovascular diseases (for mortality evaluation), prescribing of antidiabetic, antihypertensive, statins and antiplatelet drugs, and index year.

Figure 2. Relative hazards of presence of chronic kidney disease (CKD) for mortality and cardiovascular events compared with non-CKD as reference. Hazard ratios (HRs) for all-cause mortality and subdistribution hazard ratios (SHRs) for cardiovascular mortality, coronary heart disease, and stroke were adjusted for age, gender, body mass index, deprivation level, duration of diabetes, proteinuria status, a history of cardiovascular diseases (for mortality evaluation), prescribing of antidiabetic, antihypertensive, statins and antiplatelet drugs, and index year.

For peer review only

For peer review only - http://bmjopen.bmj.com/site/about/guidelines.xhtml

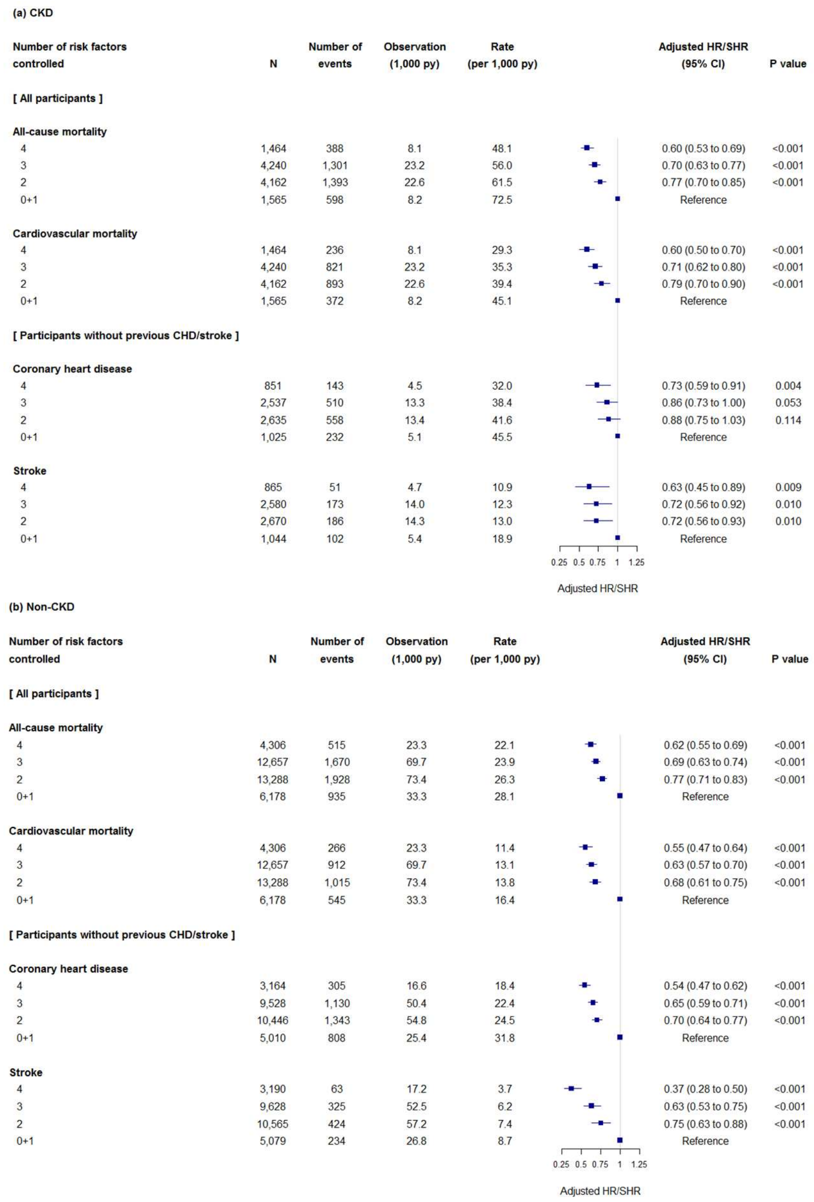

figure1

105x156mm (300 x 300 DPI)

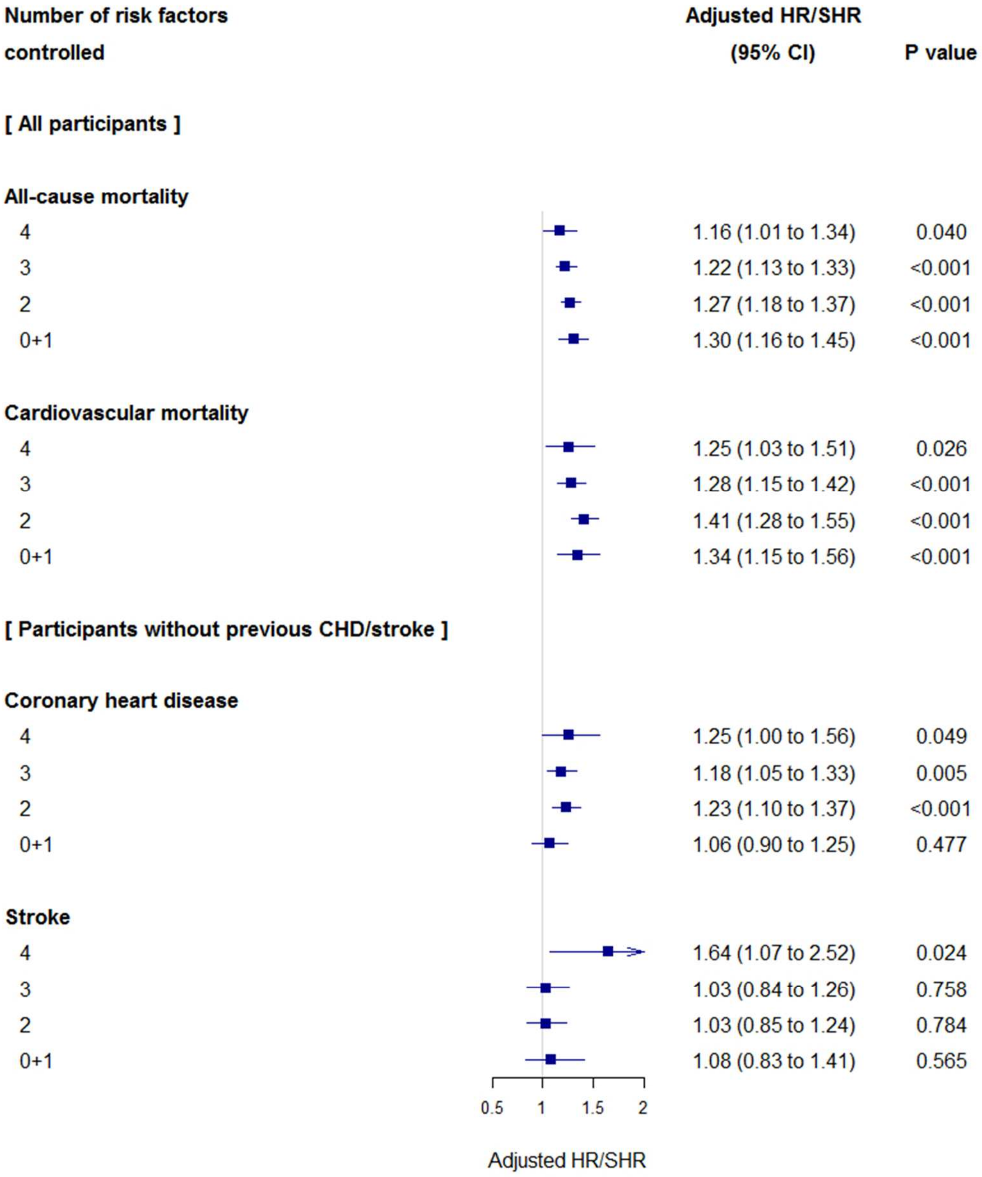

figure2

66x78mm (300 x 300 DPI)

SUPPLEMENTARY MATERIAL

Figure S1. Study cohort selection

Potentially eligible participants with diabetes between 2006 and 2010 (n=279,182)

↓ →

- Participants who were diagnosed with type 1 or other specific types of diabetes
- Participants who were first diagnosed with diabetes before 30 years of age
- Participants who were first prescribed insulin within 180 days of the first diagnosis

↓

Participants with type 2 diabetes (n=233,698)

↓

Participants with a valid serum creatinine measurement between 2006 and 2010 (n=176,311)

↓

Participants confirmed with prevalent diabetes using data within 12 months before the index date (n=127,792)

↓

Participants aged 40 to 79 years at index date (n=108,502)

↓

Participants with data at least 1 year before and after index date (n=106,799)

↓

Participants with linked data for hospital care and death registration (n=66,100)

↓ →

- Participants with a diagnosis of ESRD/RRT at baseline
- Participants with index eGFR <15 mL/min/1.73 m^2

↓

For peer review only

For peer review only - http://bmjopen.bmj.com/site/about/guidelines.xhtml

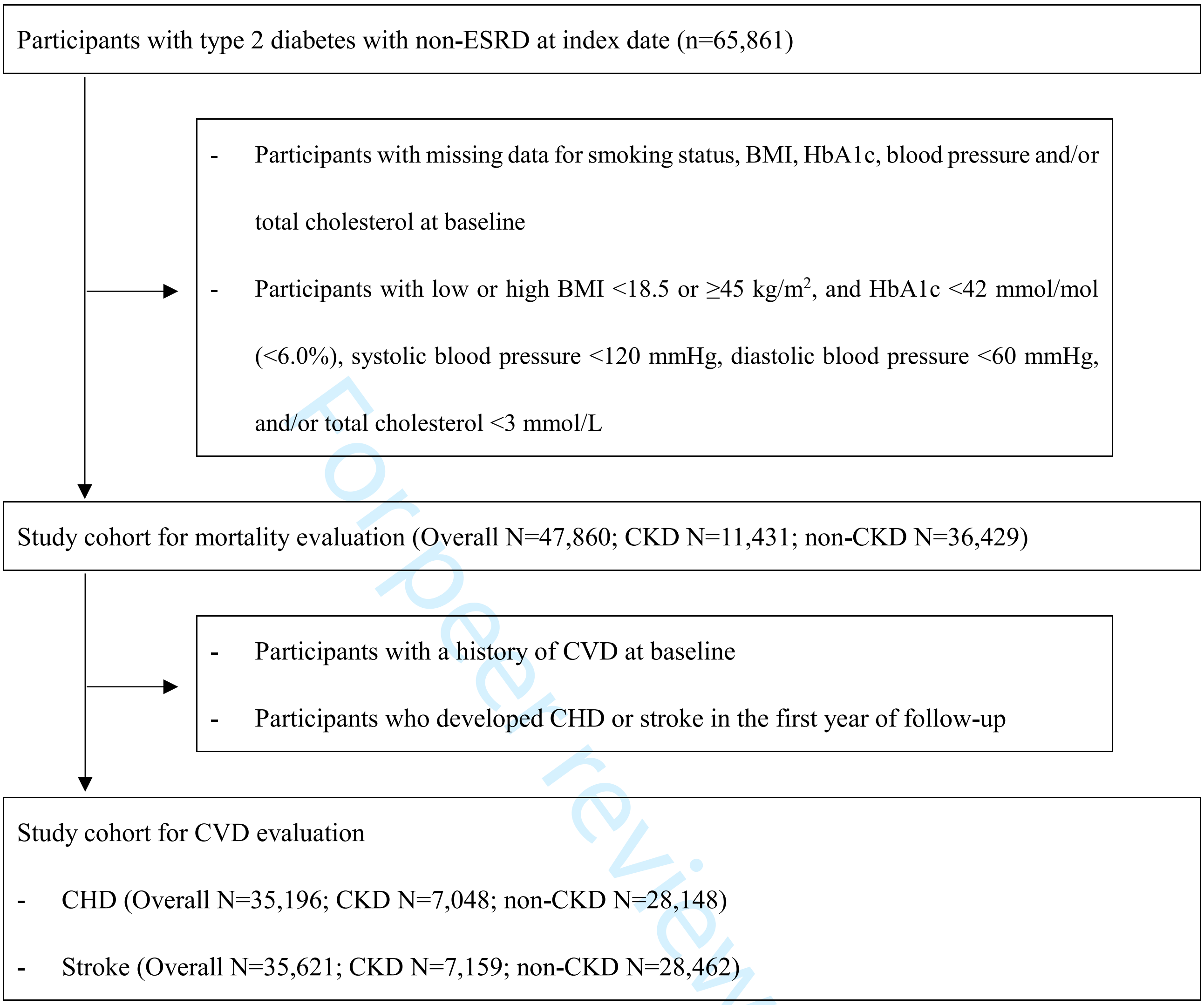

BMI, body mass index; CKD, chronic kidney disease; CHD, coronary heart disease; CVD, Cardiovascular diseases; eGFR, estimated glomerular filtration rate; ESRD, end-stage renal disease; RRT, renal replacement therapy

For peer review only

For peer review only - http://bmjopen.bmj.com/site/about/guidelines.xhtml

Table S1. Breakdown of risk factors controlled according to chronic kidney disease and a history of cardiovascular diseases

Number of risk factors controlled	HbA1c <53 mmol/mol (<7.0%)	Blood pressure <140/90 mmHg	Total cholesterol <5 mmol/L	No smoking	CKD			Non-CKD		
					Overall (N=11,431)	No CVD (N=7,216)	CVD (N=4,215)	Overall (N=36,429)	No CVD (N=28,569)	CVD (N=7,860)
0	–	–	–	–	138 (1)	87 (1)	51 (1)	806 (2)	678 (2)	128 (2)
1	Y	–	–	–	69 (0.6)	46 (0.6)	23 (0.6)	312 (1)	260 (0.9)	52 (0.7)
	–	Y	–	–	89 (0.8)	64 (0.9)	25 (0.6)	759 (2)	624 (2)	135 (2)
	–	–	Y	–	438 (4)	264 (4)	174 (4)	1,742 (5)	1,350 (5)	392 (5)
	–	–	–	Y	831 (7)	597 (8)	234 (6)	2,559 (7)	2,187 (8)	372 (5)
2	Y	Y	–	–	62 (0.5)	43 (0.6)	19 (0.5)	304 (0.8)	268 (0.9)	36 (0.5)
	Y	–	Y	–	245 (2)	152 (2)	93 (2)	794 (2)	590 (2)	204 (3)
	Y	–	–	Y	407 (4)	289 (4)	118 (3)	1,070 (3)	911 (3)	159 (2)
	–	Y	Y	–	390 (3)	215 (3)	175 (4)	1,911 (5)	1,446 (5)	465 (6)
	–	Y	–	Y	531 (5)	379 (5)	152 (4)	2,205 (6)	1,910 (7)	295 (4)
	–	–	Y	Y	2,527 (22)	1,615 (22)	912 (22)	7,004 (19)	5,477 (19)	1,527 (19)
3	Y	Y	Y	–	247 (2)	152 (2)	95 (2)	1,073 (3)	788 (3)	285 (4)
	Y	Y	–	Y	302 (3)	199 (3)	103 (2)	1,067 (3)	905 (3)	162 (2)
	Y	–	Y	Y	1,552 (14)	1,019 (14)	533 (13)	3,487 (10)	2,678 (9)	809 (10)
	–	Y	Y	Y	2,139 (19)	1,228 (17)	911 (22)	7,030 (19)	5,294 (19)	1,736 (22)
4	Y	Y	Y	Y	1,464 (13)	867 (12)	597 (14)	4,306 (12)	3,203 (11)	1,103 (14)

Y, meeting the criterion; –, not meeting the criterion

CKD, chronic kidney disease; CVD, (a history of) cardiovascular diseases

For peer review only

Figure S2. Relative hazards of individual risk factors controlled for mortality and cardiovascular events in (a) participants with chronic kidney disease (CKD) and (b) participants with non-CKD. Hazard ratios (HRs) for all-cause mortality and subdistribution hazard ratios (SHRs) for cardiovascular mortality, coronary heart disease, and stroke were adjusted for age, gender, CKD stage (for CKD cohort), body mass index, deprivation level, duration of diabetes, proteinuria status, a history of cardiovascular diseases (for mortality evaluation), prescribing of antidiabetic, antihypertensive, statins and antiplatelet drugs, and index year.

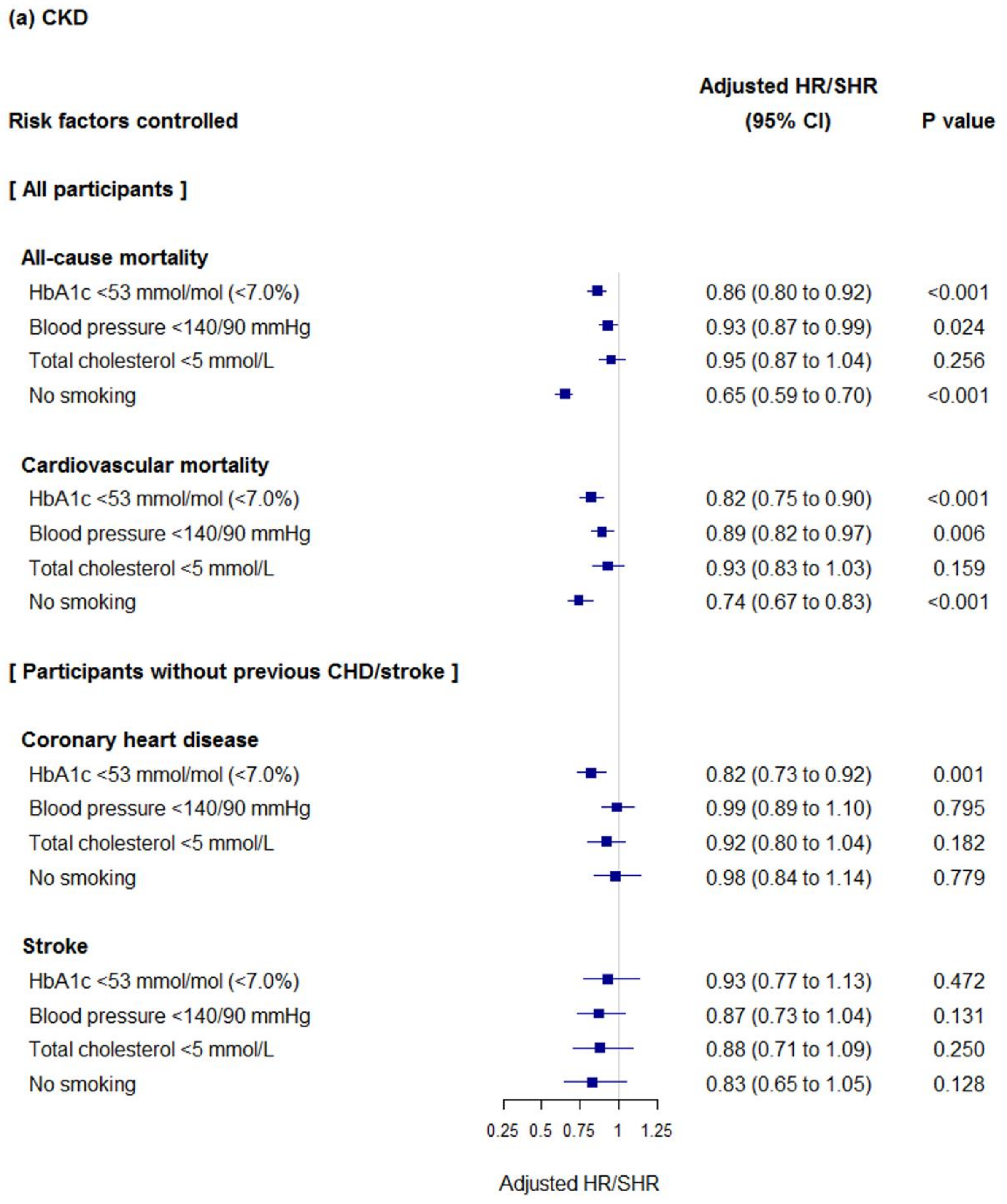

For peer review only - http://bmjopen.bmj.com/site/about/guidelines.xhtml

(b) Non-CKD

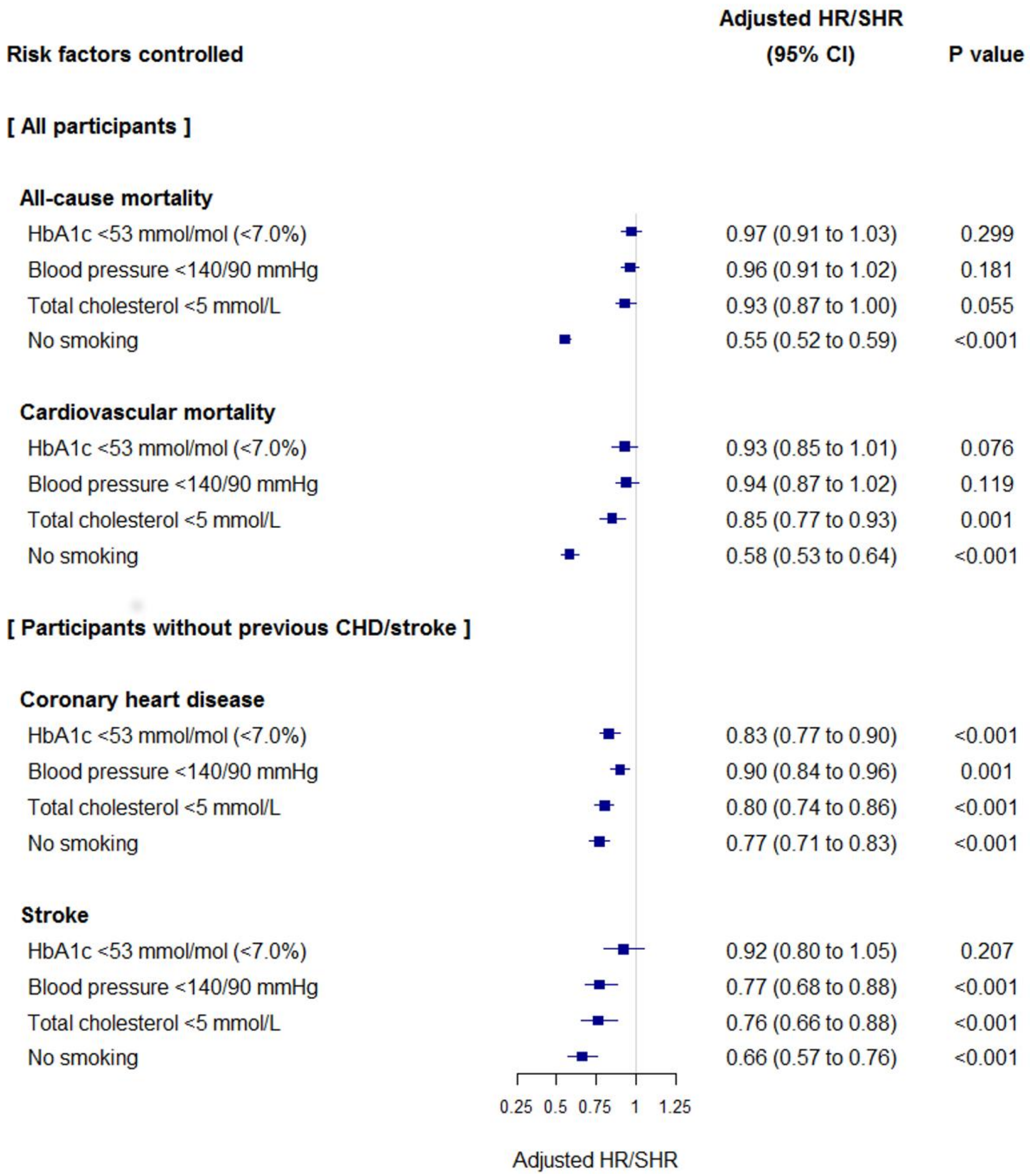

For peer review only - http://bmjopen.bmj.com/site/about/guidelines.xhtml

STROBE 2007 (v4) Statement—Checklist of items that should be included in reports of *cohort studies*

Section/Topic	Item #	Recommendation	Reported on page #
**Title and abstract**	1	(*a*) Indicate the study's design with a commonly used term in the title or the abstract	1,2
		(*b*) Provide in the abstract an informative and balanced summary of what was done and what was found	2,3
**Introduction**			
Background/rationale	2	Explain the scientific background and rationale for the investigation being reported	5,6
Objectives	3	State specific objectives, including any prespecified hypotheses	6
**Methods**			
Study design	4	Present key elements of study design early in the paper	6,7
Setting	5	Describe the setting, locations, and relevant dates, including periods of recruitment, exposure, follow-up, and data collection	6,7
Participants	6	(*a*) Give the eligibility criteria, and the sources and methods of selection of participants. Describe methods of follow-up	7,8,10
		(*b*) For matched studies, give matching criteria and number of exposed and unexposed	NA
Variables	7	Clearly define all outcomes, exposures, predictors, potential confounders, and effect modifiers. Give diagnostic criteria, if applicable	9
Data sources/ measurement	8*	For each variable of interest, give sources of data and details of methods of assessment (measurement). Describe comparability of assessment methods if there is more than one group	6-9
Bias	9	Describe any efforts to address potential sources of bias	10,11
Study size	10	Explain how the study size was arrived at	6-8
Quantitative variables	11	Explain how quantitative variables were handled in the analyses. If applicable, describe which groupings were chosen and why	11
Statistical methods	12	(*a*) Describe all statistical methods, including those used to control for confounding	11
		(*b*) Describe any methods used to examine subgroups and interactions	11
		(*c*) Explain how missing data were addressed	8,11
		(*d*) If applicable, explain how loss to follow-up was addressed	10
		(*e*) Describe any sensitivity analyses	NA
**Results**			

For peer review only

Participants	13*	(a) Report numbers of individuals at each stage of study—eg numbers potentially eligible, examined for eligibility, confirmed eligible, included in the study, completing follow-up, and analysed	12,Figure S1
		(b) Give reasons for non-participation at each stage	12,Figure S1
		(c) Consider use of a flow diagram	Figure S1
Descriptive data	14*	(a) Give characteristics of study participants (eg demographic, clinical, social) and information on exposures and potential confounders	12,13,Table 1
		(b) Indicate number of participants with missing data for each variable of interest	Table 1,Figure S1
		(c) Summarise follow-up time (eg, average and total amount)	Figure 1
Outcome data	15*	Report numbers of outcome events or summary measures over time	Figure 1
Main results	16	(*a*) Give unadjusted estimates and, if applicable, confounder-adjusted estimates and their precision (eg, 95% confidence interval). Make clear which confounders were adjusted for and why they were included	12-15
		(*b*) Report category boundaries when continuous variables were categorized	9,11
		(*c*) If relevant, consider translating estimates of relative risk into absolute risk for a meaningful time period	NA
Other analyses	17	Report other analyses done—eg analyses of subgroups and interactions, and sensitivity analyses	NA
**Discussion**			
Key results	18	Summarise key results with reference to study objectives	15-16
**Limitations**			
Interpretation	20	Give a cautious overall interpretation of results considering objectives, limitations, multiplicity of analyses, results from similar studies, and other relevant evidence	15-18
Generalisability	21	Discuss the generalisability (external validity) of the study results	17,18
**Other information**			
Funding	22	Give the source of funding and the role of the funders for the present study and, if applicable, for the original study on which the present article is based	19

*Give information separately for cases and controls in case-control studies and, if applicable, for exposed and unexposed groups in cohort and cross-sectional studies.

Note: An Explanation and Elaboration article discusses each checklist item and gives methodological background and published examples of transparent reporting. The STROBE checklist is best used in conjunction with this article (freely available on the Web sites of PLoS Medicine at http://www.plosmedicine.org/, Annals of Internal Medicine at http://www.annals.org/, and Epidemiology at http://www.epidem.com/). Information on the STROBE Initiative is available at www.strobe-statement.org.

For peer review only
